# Restoration of the reduced CLSP activity alleviates memory impairment in Alzheimer disease

**DOI:** 10.1038/s41398-020-01168-8

**Published:** 2021-01-13

**Authors:** Yuichi Hashimoto, Shinya Kusakari, Mikiro Nawa, Koichi Okamoto, Yuka Toyama, Masaaki Matsuoka

**Affiliations:** 1 Department of Pharmacology, Tokyo Medical University, 6-1-1 Shinjuku, Shinjuku-ku, Tokyo, 160-8402 Japan; 2 Department of Neurology, Geriatrics Research Institute and Hospital, Maebashi, Gunma Japan

**Keywords:** Drug discovery, Molecular neuroscience

## Abstract

Calmodulin-like skin protein (CLSP), a secreted peptide, inhibits neuronal death in cell-based Alzheimer’s disease (AD) models and transgenic overexpression of the *CLSP* gene suppresses synaptic loss and memory impairment in AD model mice, *APPswe/PS1dE9* double transgenic mice (APP/PS1 mice). Despite the anticipated role of CLSP as an AD-suppressing factor, it remains unanswered whether the insufficiency of the CLSP activity is linked to the AD pathogenesis. In this study, we first show that adiponectin, a CLSP potentiator/protector, dominantly determines the CLSP activity in the central nervous system where there are sufficient concentrations of CLSP, higher concentrations of CLSP inhibitors such as apolipoprotein E, and smaller concentrations of adiponectin. We next show that both the levels of brain adiponectin and the intraneuronal levels of SH3BP5, an important effector of the CLSP signal, are reduced in both AD patients and APP/PS1 mice. Finally, the restoration of the CLSP activity by subcutaneous injection of a hybrid peptide named CLSPCOL consisting of CLSP(1-61) and the collagen-homologous region of adiponectin, which has more potent neuroprotective activity than CLSP, is insensitive to the suppression by the CLSP inhibitors, and is efficiently recruited into brains, alleviates dementia and synaptic loss in the aged APP/PS1 mice. Collectively, these results suggest that the reduction in the CLSP activity, likely caused by the reduction in the levels of adiponectin, leads to the insufficient protection of neurons from neurotoxicity in the AD brains and the restoration of the CLSP activity is a promising strategy for the treatment of AD.

## Introduction

Alzheimer’s disease (AD) is a major neurodegenerative disease that causes dementia. The AD pathogenesis is still under investigation, and disease-modifying therapy has not been available for the AD treatment^1–3^.

The secreted peptides, namely, humanin and calmodulin-like skin protein (CLSP), are physiological agonists for the heterotrimeric humanin receptor (htHNR), consisting of ciliary neurotrophic factor α, WSX-1, and gp130^4–6^. They inhibit AD-related neuronal cell death in vitro^5,7^, and the transgenic overexpression of the mouse *CLSP-1* gene protects against synaptic loss and memory loss in AD model mice, *APPswe/PS1dE9* double transgenic mice (APP/PS1 mice), in an amyloid β (Aβ)-independent fashion^8^. A previous study has suggested that humanin is expressed in astroglial cells and involved in neuroprotection via the humanin receptor^9^. However, the activity of humanin is weak (50% effective concentration is approximately 0.1–1 μM) and the in vivo concentration of humanin appears insufficient to exert the neuroprotective effect^10^. Moreover, it is highly likely that humanin inhibitors work in the brains^11^.

CLSP is mainly produced in the skin keratinocytes and to a less extent, in epithelial cells of some peripheral tissues^12–14^ and likely reaches the central nervous system (CNS) via blood circulation^5,15,16^. CLSP is 10^4^-fold more potent than humanin (50% effective concentration is approximately 0.1 nM)^5^. Since the concentration of CLSP in the CNS is sufficient to exert the neuroprotective effect^16^, CLSP, rather than humanin, plays a central role in the activation of the htHNR in vivo^5,6,10,16^. Given CLSP is a putative AD-suppressing factor, it could be hypothesized that concentrations of CLSP are reduced in the CNSs of AD patients. However, our previous study showed that they were not reduced in the cerebrospinal fluids (CSFs) of AD patients^16^.

CLSP interacts with multiple proteins^17^, although it remains unknown whether they affect the function of CLSP. In the current study, we first unraveled the function of the CLSP interactors. Using samples from AD patients and aged APP/PS1 mice, we next investigated whether the CLSP activity, which is dominantly regulated by the adiponectin level and likely correlates with the intraneuronal SH3BP5 level, was reduced in the CNS of AD. Finally, we examined whether the restoration of the reduced CLSP activity by subcutaneous injection of a hybrid peptide named CLSPCOL, consisting of CLSP(1-61) and the collagen-homologous region of adiponectin, alleviated dementia and synaptic loss in the aged APP/PS1 mice.

## Materials and methods

### Mouse handling and administration of CLSP

All mice used in the current study were male. All animal experiments were approved by the Institutional Animal Care and Use Committee of Tokyo Medical University (No. H290026) and were conducted in accordance with the Society’s Policies on the Use of Animals in Neuroscience Research. The numbers of mice and mouse ages used in an experiment are presented in each figure legend. APP/PS1 mice (B6C3-Tg(APPswe,PSEN1dE9)85Dbo/\Mmjax, RRID:MMRRC_034832 JAX) were obtained from the Jackson Laboratory (Bar Harbor, ME). After weaning at one month of age, mice were genotyped by tail specimens and identified by ear punched IDs. Four mice from each genotype line were arbitrarily selected and housed into a cage according to the descending order of ID number, and totally eight mice from two genotype lines were housed together. Mice were allocated to all experiments according to the descending order of ID number as well as housing. The experimenters were not blinded to the genotypes of mice which they were handling. Mice were bred and maintained at the Pre‐clinical Research Center of Tokyo Medical University under specific pathogen free conditions. They were housed in an air conditioned room with a 12/12 h dark/light cycle. Mouse CLSPCOL (mCLSPCOL) in saline or distilled water was subcutaneously injected to male APP/PS1 and littermate wt mice with an age of 16 months. 5 nanomol of mCLSPCOL was injected everyday until sacrifice for histological analysis. For pharmacokinetics and pharmacodynamics analysis, 5 nanomol of mCLSPCOL was once subcutaneously injected. In an experiment, mCLSPCOL was similarly intranasally administered, as described previously^18^.

### Human cerebrospinal fluids (CSFs) and temporal lobe samples for ELISA

All experiments were approved by the Institutional Human Ethics Committees of Tokyo Medical University. Postmortem CSF and temporal lobe samples from AD patients and controls for ELISA assays were obtained from the Kathleen Price Bryan Brain Bank at the Division of Neurology, Duke University Medical Center. Pathological staging was performed under “The Consortium to Establish a Registry for AD” (CERAD) staging system for senile and neuritic plaques^19^ and under the Braak staging systems for neurofibrillary tangles^20^. Possible AD cases defined by the CERAD staging were counted as AD cases. The study was also approved by the local ethics committees of the Kathleen Price Bryan Brain Bank at the Division of Neurology, Duke University Medical Center.

### Preparation of interstitial fluid (ISF)-containing brain samples and cell-broken brain lysates after the subcutaneous injection of CLSPCOL to mice

At 1 h or 25 h after the last injection, mice were anesthetized with diethyl ether (Wako Pure Chemicals, Tokyo, Japan). Blood was then aspirated from hearts and centrifuged at 4000 × g for 10 min at 4 °C. The vascular space of the brain was washed to be free of blood by the perfusing 20 ml of ice-cold lactated-Ringer’s solution (Otsuka Pharmaceuricals, Tokyo, Japan) through the left ventricle of the heart. Subsequently, the mice were decapitated and the brain were removed. The whole brain was once washed with the lactated-Ringer’s solution, to be free of the contamination of CSF. The cortex was then separated into the right and left hemispheres. The left hemisphere was then homogenized with two-fold weight of saline. After the homogenate was centrifuged at 4000 × g for 10 min at 4 °C, the supernatant was collected as the three-times diluted ISF-containing brain sample. The right hemisphere was suspended in 50 mM HEPES (pH7.4), 150 mM NaCl, 0.1% NP-40, and protease inhibitor cocktail Complete (Roche Diagnostics, Basel, Switzerland). After freezing and thawing twice, the brain lysate was centrifuged at 15,000 rpm for 10 min at 4 °C to obtain the supernatants for the cell-broken brain lysate. The ISF-containing samples and the cell-broken brain lysates were submitted to ELISA analysis.

### Measurement of dissociation constants

The dissociation constant for the binding between apolipoprotein (ApoE) 4 (or adiponectin) and CLSP was measured using the Nano-Glo HiBiT Extracellular Detection System (cat. no.: N2430, Promega, Madison, WI). For the coating of recombinant ApoE4 or adiponectin, 100 μl of 50 mM carbonate buffer (pH 9.6) containing 20 pM of ApoE4 or adiponectin was incubated for overnight at 4 °C in the wells of the 96-well plates (Fluorescence-use Black-type Plate H, cat. no.: MS-8596KZ, Sumitomo Bakelite, Tokyo, Japan). The protein-coated plates were washed three times with 200 μl of PBS. Then, 150 μl of PBS containing 1% skim milk (GIBCO) was added to each well. They were incubated for 1 h at room temperature without shaking. After the plates were washed three times with 200 μl of PBS, 100 μl of a certain concentration of CLSP-HiBiT in PBS was added to each well. The plates were further incubated for overnight at 4 °C without shaking and then washed five times with PBS containing 0.1% NP-40, followed by the addition of 100 μl of PBS. Then, the substrate for HiBiT in the kit was added to each well. Resulting chemiluminescence was measured for each well using by Wallac ARVO^TM^ X5 (Perkin Elmer). The CLSP-HiBiT concentration was estimated for each well, referring to a standard curve or line that was simultaneously obtained by measuring chemiluminescence of wells that were filled with 100 μl of PBS containing stepwise increasing concentrations of recombinant CLSP-HiBiT. This experiment was performed in duplicate.

### ELISA

Experimenters were blinded to the identities of human samples in ELISA. The peroxidase-labelled sigma-C, SH3BP5, or mouse adiponectin antibody was prepared using Ab-10 Rapid Peroxidase Labeling Kit (cat. no.: LK33, Dojindo, Kumamoto, Japan) or Peroxidase Labeling Kit-HN_2_ (cat. no.: LK33, Dojindo). For 14-3-3σ and SH3BP5 ELISA, 100 μL of 50 mM of carbonate buffer (pH9.6) containing 0.6 μg/ml of the GST-sigma antibody or 1 μg/ml of a SH3BP5 monoclonal antibody (clone 1D5, cat. no.: H00009467-M02, Abnoba, Taipei, Taiwan) as a capture antibody was incubated for overnight at 4 °C in a 96-well plate (ELISA Plate H, cat. no.: MS-8896FZ, Sumitomo Bakelite, Tokyo, Japan). The plates were washed three times with 400 μl of the wash buffer (PBS containing 0.1% NP-40) and filled with 300 μl of PVDF Blocking Reagent (cat. no.: NYPBR01, TOYOBO, Tokyo, Japan) for 1 h at room temperature without shaking. After washed three times with 300 μl of the wash buffer, the plates were filled with 100 μl of stepwise-increasing concentrations of recombinant 14-3-3σ or SH3BP5 in PBS (for the measurement of a standard curve or line), human CSF samples or the cell-broken lysates of human temporal lobes, and incubated for 2 h at room temperature with shaking at 250 rpm. Then, the plates were washed with 300 μl of the wash buffer. A 100 μl of 1.0 μg/ml detection antibody (the peroxidase-labelled sigma-C or SH3BP5 antibody-GST-SH3BP5) in Can Get Signal Solution 2 (cat. no.: NKB-301, TOYOBO) was added to each well, and the plates were incubated for 1 h at room temperature with shaking (250 rpm). SH3BP5 ELISA can measure both mouse and human SH3BP5 almost to an equal extent.

Ready-made ELISA kits for human and mouse adiponectin were purchased from Sekisui Medical Co., Ltd (cat. no.: 376405, Tokyo, Japan) and RayBioTech (Peachtree Corners, GA). Ready-made ELISA kits for human and mouse ApoE were purchased from Abcam (cat. no.: ab108813 and ab215086, Cambridge, UK). Ready-made ELISA kits for human Aβ1-40, Aβ1-42, and soluble Aβ oligomers (more than 10 mers) were purchased from Wako Pure Chemicals (cat. no.: 298‐62301, 298‐62401, and 298-80101).

Single-step ELISA systems for mCLSPCOL and mouse CLSP were generated as follows. Ninety-sixth well plates (ELISA Plate H, cat. no.: MS-8896FZ, Sumitomo Bakelite, Tokyo, Japan) were filled with 100 μl of 1 μg/ml streptavidin, purchased from New England Biolabs. (cat. no.: N7021, Ipswich, MA), in PBS and incubated overnight. After blocked with PVDF Blocking Reagent (TOYOBO) for 1 h at 25 °C and washed, the 96-well plates were filled with 100 μL of PBS containing 25 μg/ml of the biotin-conjugated mouse CLSP antibody, diluted ISF-containing samples, and peroxidase-conjugated mouse adiponectin antibody and incubated for 1 h at 25 °C with shaking at 100 rpm. To make a standard curve or line, 100 μl of stepwise-increasing concentrations of mCLSPCOL and recombinant mouse CLSP-1 in PBS were filled in place of ISF-containing samples.

After washed five times with 300 μl of the wash buffer, each well was filled with R&D TMB Substrate solution (cat. no.: DY999, R&D Systems), and the plates were incubated for 10 min at room temperature. The reaction was stopped by the addition of 50 μl of 2N H_2_SO_4_. Absorbance at 450 nm was measured using Wallac ARVO^TM^ X5.

### Immunohistochemical analysis of human and mouse samples

Histological brain samples were obtained at Gunma Geriatric Research Hospital under established procedures after obtaining a written informed consent from the family of each patient. Patients were diagnosed as having AD using clinical criteria and the diagnosis was confirmed by neuropathological analysis at autopsy. At autopsy, brains were fixed with 4% paraformaldehyde in PBS (pH 7.4), embedded in paraffin, and then subjected to neuropathological examination. Cerebral cortices and hippocampi used in this study originated from samples of 7 patients with sporadic AD and 6 patients with sporadic amyotrophic lateral sclerosis (ALS), a representative motoneuron-specific neurodegenerative disease. The brains of ALS patients were used as negative controls because the neurodegeneration occurs only in the motor area of the temporal lobe in ALS.

Sliced sections were deparaffinized, rehydrated to PBS, and unmasked in ANTIGEN UNMASKING SOLUTION (Vector Laboratories, Burlingame, CA) at 70 °C for 15 min. Subsequently, the sections were incubated at room temperature for 20 min in a blocking solution containing goat normal serum and 0.3% Triton X-100 in TBS, and then incubated at 4 °C for three and more overnights with 5 μg/ml mouse IgG1 as negative control (cat. no.: MAB002, R&D Systems, Minneapolis, MN) or SH3BP5 (Sab) monoclonal antibody clone PL-A23 (cat. no.: sc-135617, Santa Cruz Biotech., Santa Cruz, CA) in PBS containing 1% BSA. Immunoreactivity was visualized using TSA (Tyramide Signal Amplification)-Plus Fluorescein System (Perkin-Elmer, Waltham, MA) (Tyramide-Red method). Fluorescence-labeled samples were observed with a fluorescence microscope (Biozero BZ-8000, KEYENCE, Osaka, Japan). Fluorescence images were analyzed using NIH Image J 1.37 v.

For obtaining tissues for histological analysis, mice were first anesthetized by inhalation of isoflurane, and then perfused transcardially with PBS containing 10 unit/ml heparin sodium (Mochida pharmaceutical co., ltd., Tokyo, Japan) and then with the fixation buffer [4% paraformaldehyde in 0.1 M phosphate buffer (pH 7.4)]. Brains were dissected and fixed again in the fixation buffer and incubated overnight at 4 °C with gentle shaking. These tissues were subsequently transferred to sucrose solution (30% [w/v] in 0.1 M phosphate buffer [pH 7.4]), embedded in OCT compound (Sakura Fine Technical, Tokyo, Japan), and rapidly frozen in liquid nitrogen. Frozen sections with a thickness of 10 μm were prepared with a cryostat, mounted on MAS-coated glass slides (Matsunami Glass Ind., Ltd., Osaka, Japan), and air-dried. All sections were then incubated for 1 h at 22 °C in blocking buffer (PBS supplemented with 5% BSA and 0.1% Triton X-100). They were stained two overnight at 4 °C with primary antibodies diluted in the blocking buffer. They were then washed with PBS and exposed to corresponding secondary antibodies conjugated with the fluorescent dye Cy3 (Jackson Immuno Research Laboratories, West Grove, PA). Fluorescence images were acquired using an LSM710 confocal microscope (Carl Zeiss, Oberkochen, Germany). PSD95‐positive puncta were counted in three representative 50 × 50 μm areas in the hippocampal CA1 regions and the average number of puncta per area was calculated. The mean fluorescence intensity of synaptophysin in a 100 × 100 μm area of the hippocampal CA1 or CA3 region was measured using the ZEN software.

### Quantification of SH3BP5 immunofluorescence intensity in neurons

Using NIH Image J 1.37 v, the SH3BP5 immunofluorescence intensity and the area of a selected neuron were quantified. Mean SH3BP5 immunofluorescence intensity per one μm^2^ (a) of the neuron was calculated. Mean immunofluorescence intensity per one μm^2^ of the neuropil around the neuron was simultaneously quantified as a background immunofluorescence (b). The subtracted mean immunofluorescence intensity (a-b) was the mean SH3BP5 immunofluorescence intensity of the neuron. Then, the a-b value was multiplied by the neuronal area to estimate the level of SH3BP5 expression in the neuron. Ten neurons were selected at random. The average immunofluorescence intensity in 10 neurons per sample was calculated for each sample.

### Modified Morris water maze test and Y-maze test

Sample sizes were determined according to the previous study^8^. Animals were not allocated to groups by randomization. Experimenters were not blinded to mouse groups. Male APP/PS1 mice and their littermate wt mice were once subjected to a modified Morris water maze test (8) at age of 13 months for independent purposes. At ages of 16 months, they were subcutaneously injected once per day with 5 nmol of mCLSPCOL or saline for 18 days. Morris water maze tests were started on the eighth day. Mice were placed in a water bath and trained to escape onto the platform in the pool for three consecutive days (8^th^ to 10^th^ days; 14-15 male mice per each group). The pool was 100 cm in diameter and filled with opaque water at 22 °C. A platform (10 cm in diameter) was used for the hidden platform trials, and the top surface of the platform was 1 cm below the water surface. The time spent to find and escape onto the hidden platform was measured. The first trial was started at 9:30 AM on each day, and three trial sessions per day were performed with an interval of about 30 min. In each training session, mice were allowed to swim until they found the platform or until 60 s had elapsed. Mice that failed to find the platform were guided to the platform and then allowed to remain there for 30 s. The behavior of mice was recorded with a CCD camera, and the total traveled distance, average swimming speed, escape latency to the platform, and time spent in the target quadrant were measured automatically (O’Hara & Co., Tokyo, Japan). A wt mouse was excluded from analysis because it did not move in this experiment. Twenty four hours after the first training session, the platform was removed, and a probe test was performed for 60 s on the 11^th^ day.

After the water maze test was finished, spatial working memory performance was evaluated by monitoring spontaneous alternation behavior in the Y maze test. The Y maze test was carried out as described previously^21^, from the 16^th^ to 18^th^ days. Briefly, the Y maze consists of three intersecting black acrylic arms. Each arm is 40 cm long, whose cross section has a trapezoidal shape, a top with 10 cm, a bottom with 3 cm, and a height of 12 cm. Each mouse was placed at the end of one arm and allowed to explore the Y maze freely for 8 min, and the series of arm entries were recorded to evaluate short‐term/spatial working memory by spontaneous alternation behavior. Mice were considered to have chosen an arm when their entire torsos had reached the arm. Spontaneous alternation behavior is based upon the behavior of animals in preferring to choose newer locations. When mice chose a third arm different from the previous two in the consecutive three arm choices in the Y maze, the mice were considered to have carried out actual alternation. Alternation behavior (%) was calculated as the percentage of actual alternations to maximum alternations that were defined as the total number of arm entries minus two. For example, if a mouse made 10 entries in an order such as Arm A-B-C-B-A-B-C-B-A-C, the actual and maximum alternation would be 5 and 8, respectively, and alternation behavior would be 62.5%. The numbers of arm entries were also counted.

### Inclusion and exclusion criteria

Assay results for all the samples in the cell death assays were used for statistical analysis. If CSF samples were apparently contaminated with blood, they were excluded from assays. To exclude the effect of sexual cycle on the results, only male mice were used for all experiments using mice. If mice did not move at all, they were excluded from analysis in the Morris water maze test. In the Y maze test, if the number of the arm entries of a mouse was less than six times, it was excluded from the analysis.

### Statistical analysis

All data were analyzed using Prism7 for Mac OSX software (GraphPad, San Diego, CA). Data in all cell-death experiments are shown as means ± standard deviation (SD). All the other data are indicated as means ± standard error of the means (SEM). Statistical analysis was performed using one-way or two-way ANOVA analysis, followed by *post hoc* Tukey’s or Dunnett’s multiple comparison test. Unpaired *t*-test (two-tailed) was used for the analysis of data, if experiments contained only two groups. If *p*-value was less than 0.05, it was regarded as statistically significant.

### Genes and vectors

### Immunoblot analysis

### Pull-down analysis

### Recombinant proteins

### Mouse CLSPCOL

### Preparation of lysates for the measurement of Aβ

### Cell death assays

### Antibodies

All these procedures and materials are described in detail in Supplementary information.

## Results

### Characterization of CLSP inhibitors, including apolipoprotein E

**Fig. 1:**
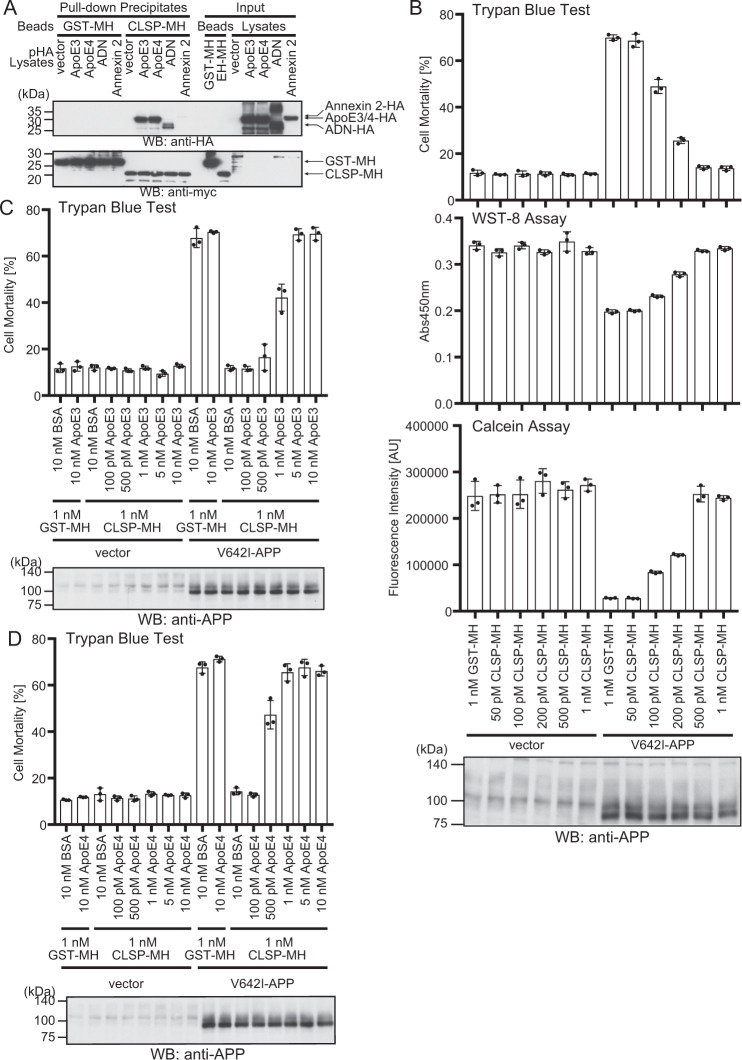
Apolipoproteins E suppresses CLSP activity. **a** ApoE3, E4, adiponectin (ADN), and annexin 2, C-terminally tagged with HA, were overexpressed in F11 neurohybrid cells. Resulting cell lysates were mixed with bacterially produced GST-MycHis- or CLSP-MycHis-conjugated sepharose 4B and incubated at 4 °C overnight. The pulled-down precipitates were immunoblotted using HA and myc antibodies. For reference, inputs including the sepharose 4B beads conjugating GST-MycHis (GST-MH) and CLSP-MycHis (CLSP-MH) and the cell lysates were similarly immunoblotted. **b** SH-SY5Y cells, transfected with the empty vector or pcDNA3.1/MycHis-V642I-APP, were cultured in the presence of indicated concentrations of CLSP-MycHis. At 48 h, cells were harvested for trypan blue exclusion cell mortality assays (Trypan Blue Test) and cell viability assays using the WST-8 assay kit or staining with calcein AM. Cell lysates were immunoblotted using the APP antibody. **c**, **d** SH-SY5Y cells, transfected with the vector or pcDNA3.1/MycHis-V642I-APP, were cultured in media containing 1 nM of GST-MycHis or CLSP-MycHis with/without indicated concentrations of BSA, ApoE3 (**c**), or ApoE4 (**d**). At 48 h, cells were harvested for trypan blue exclusion assays. Cell lysates were immunoblotted using the APP antibody.

Multiple proteins, 14-3-3σ, 14-3-3β, calreticulin, ERp27, nucleolin, annexin 2, and annexin 5, were identified as putative CLSP interactors in a previous study (17). Independently, we have also found that apolipoprotein E (ApoE) and adiponectin bind to CLSP (Fig. 1a; Table 1).

**Table 1:** CLSP interactors and their CLSP-linked functions.

Previously found (17)
A, 14-3-3σ, 14-3-3β, calreticulin
B, annexin 2, annexin 5
C, ERp27, nucleolin
Currently found
A, apolipoprotein E
D, adiponectin

A: CLSP inhibitors, B: no effect to CLSP. C: uncharacterized, D: CLSP protector/enhancer.

Overexpression of a familial AD-causative amyloid β precursor protein (APP) mutant, V642I-APP, caused the death in SH-SY5Y neuroblastoma cells and the co-incubation with recombinant CLSP suppressed the death (Fig. 1b) (4, 5). The dose-responsive analysis revealed that the concentration of CLSP that was required to suppress neuronal death completely was 0.5 nM (Fig. 1). Interestingly, the addition of recombinant ApoE3 or ApoE4 to media suppressed the CLSP-mediated protection of the V642I-APP-induced neuronal death in a dose-responsive manner (Fig. 1c, d). Five nM of ApoE3 completely suppressed the anti-cell death effect of 1 nM of CLSP (Fig. 1c) whereas 1 nM of ApoE4 suppressed the effect of 1 nM of CLSP (Fig. 1d). This result indicates that the CLSP-inhibiting effect of ApoE4 is slightly stronger than E3. Similarly, the co-incubation with 14-3-3σ, any one of four other 14-3-3 proteins, or calreticulin completely suppressed the CLSP (10 nM)-mediated protection of the V642I-APP-induced neuronal death at the concentrations of 20-100 nM (Supplementalry Fig. 1a–f). In contrast, 100 nM of annexin 2, annexin 5, or adiponectin did not show any inhibitory action against 10 nM of CLSP at all (Supplementary Fig. 1F and Supplementary Fig. 2).

### Adiponectin keeps CLSP active in the presence of higher concentrations of CLSP inhibitors

The concentration of CLSP in human CSF was estimated to be 3–6 nM (14), which is enough for CLSP to show the neuroprotective effect. However, there seems to be much larger amounts of CLSP inhibitors in the CNS. ApoE, produced from astrocytes and microglia, is recruited into a high-density lipoprotein-like lipid in the human CNS^22,23^. Notably, the concentration of ApoE in human CSF was measured to be 40–200 nM in previous studies^24–26^. A previous study^27^ and the current study have suggested that the concentrations of 14-3-3γ and 14-3-3σ in the human CSF are lower than 1 nM (Supplementary Fig. 3). Although there are no data regarding the concentrations of the other 14-3-3 proteins in the CSF, the total expression levels of the seven 14-3-3 isoforms have been estimated to be approximately 20 times larger than that of 14-3-3γ in the brain^28^. The concentration of calreticulin in human serum was estimated to be nearly 10 pM^29^, whereas the concentration of CSF calreticulin has not been measured. In summary, the concentration of ApoE appears sufficient for ApoE to suppress CLSP function completely and the combined level of all the other inhibitors may contribute to the inhibition of the CLSP activity in vivo.

**Fig. 2:**
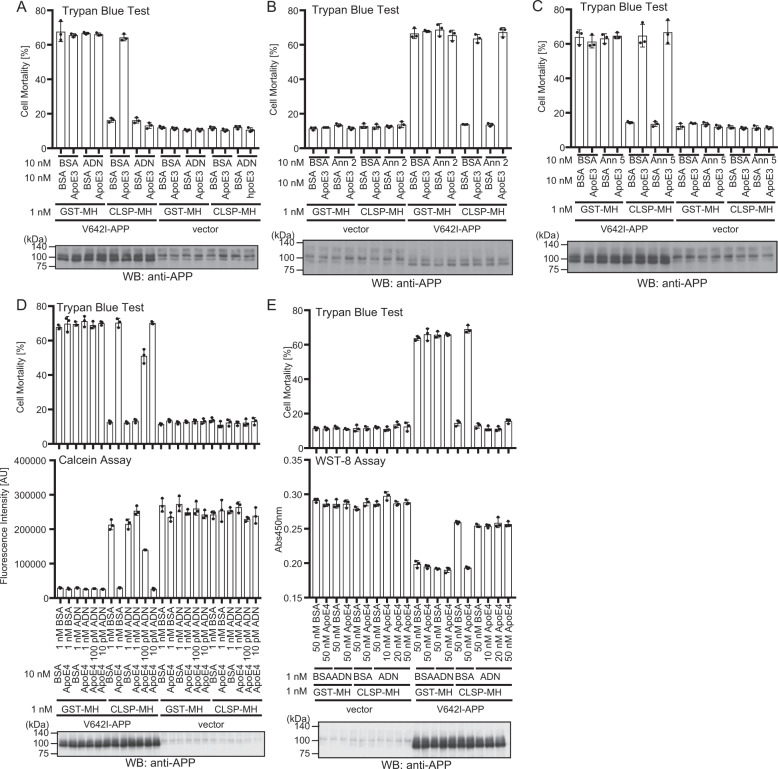
Adiponectin enables CLSP to be active in the presence of higher concentrations of apolipoprotein E4. **a**–**c** SH-SY5Y cells, transfected with the empty vector or pcDNA3.1/MycHis-V642I-APP, were cultured in media containing 1 nM of GST-MycHis or CLSP-MycHis with 10 nM of ApoE3 or BSA with 10 nM of adiponectin (ADN) (a), annexin 2 (b), annexin 5(c), or BSA. GST-MycHis and BSA were used as negative controls. At 48 h, cells were harvested for trypan blue exclusion assays. **d** SH-SY5Y cells, transfected with the vector or pcDNA3.1/MycHis-V642I-APP, were cultured in media containing 1 nM of GST-MycHis or CLSP-MycHis with/without 10 nM of ApoE4 with/without indicated concentrations of adiponectin (ADN). At 48 h, the cells were harvested for the trypan blue exclusion and calcein assays. **e** SH-SY5Y cells, transfected with the vector or pcDNA3.1/MycHis-V642I-APP, were cultured in media containing 1 nM of GST-MycHis or CLSP-MycHis with/without stepwise increasing concentrations of ApoE4 with/without 1 nM of ADN. At 48 h, the cells were harvested for trypan blue exclusion and WST-8 assays. The cell lysates were immunoblotted using the APP antibody.

Considering that there are higher amounts of CLSP inhibitors in the human brain, we next hypothesized that some CLSP interactors without CLSP-inhibiting activity might protect CLSP from the CLSP inhibitors. In reality, adiponectin (10 nM) completely nullified the ApoE3 (10 nM)-mediated inhibition of the CLSP (1 nM) activity (Fig. 2a). Neither annexin 2 nor annexin 5 showed such rescue activity at the concentrations tested (Fig. 2b, c). Dose-responsive experiments indicated that adiponectin (1 nM or 0.1 nM) completely or partially nullified the ApoE4 (10 nM)-mediated inhibition of the CLSP (1 nM) activity (Fig. 2d) and the increase in the concentrations of ApoE4 up to 50 nM did not attenuate the CLSP (1 nM)-protecting effect of adiponectin (1 nM) (Fig. 2e). Furthermore, adiponectin completely protected CLSP from the inactivation by 14-3-3σ or calreticulin (CLSP, 1 nM: 14-3-3σ or calreticulin, 2 nM or 10 nM: adiponectin, 1 nM) (Supplementary Fig. 4). These results strongly suggest that the level of adiponectin determines the CLSP activity in the CNS, where there are sufficient concentrations of CLSP (3–6 nM) and higher concentrations of the CLSP inhibitors than the CLSP concentrations.

Besides prorecting CLSP from the inactivation by the CLSP inhibitors, adiponectin may have a role in enhancing the CLSP activity. As expected, CLSP showed partial or nearly full V642I-APP-induced cell death-inhibiting effect at the concentration of 25 or 50 pM, in the presence of 200–250 pM of adiponectin (Supplementary Fig. 5A, B). Considering that CLSP alone did not inhibit V642I-APP-induced cell death at a concentration of 50 pM (Fig. 1b), we concluded that adiponectin potentiated CLSP activity. Using a recombinant trimeric adiponectin that does not form middle-molecular-weight or high-molecular-weight adiponectin spontaneously, we also showed that the trimeric adiponectin had a CLSP-enhancing effect similar to wild-type adiponectin (Supplementary Fig. S6), indicating that the multimerization^30^ that enhanced adipoectin’s metabolic activity via canonical adiponectin receptors was not essential for the CLSP-protecting effect of adiponectin.

### Adiponectin dominantly acts by binding to the endogenous humanin-like region (EHR) of CLSP that is different from the ApoE-binding region

One possible mechanism underlying the dominant CLSP-protective effect of adiponectin against ApoE is that adiponectin competes with ApoE for binding to CLSP (competitive antagonist). The pull-down assays using CLSP-conjugated sepharose 4B beads showed that the presence of adiponectin or ApoE did not greatly affect the amount of ApoE or adiponectin that were co-precipitated with CLSP (Supplementary Fig. S7A). Besides, the dissociation constant (Kd) between adiponectin and CLSP and between ApoE4 and CLSP was 7.8 and 8.8 pM, respectively (Supplementary Fig. 7B, C). These results indicate that adiponectin protects CLSP from the inactivation by ApoE, not by competing with ApoE for binding to CLSP.

**Fig. 3:**
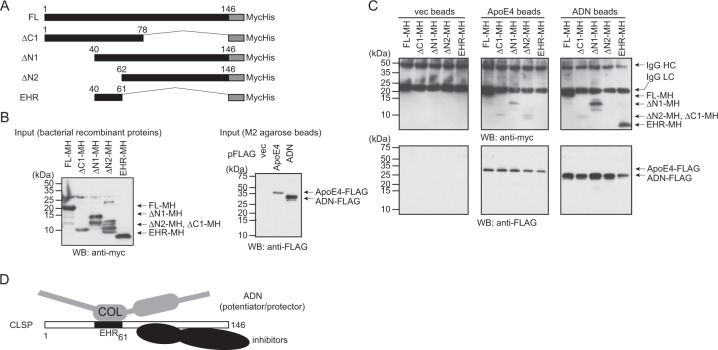
Apolipoprotein E4 and adiponectin bind to different sites of CLSP. **a** A schematic illustration of deletion mutants of CLSP. **b** Purified recombinant C-terminally MycHis-tagged CLSP (FL-MH) or CLSP deletion mutants that were produced in bacteria and C-terminally FLAG-tagged ApoE4 and adiponectin (ADN), which were purified from F11 neurohybrid cells by immunoprecipitation with FLAG antibody (M2 agarose beads), were immunoblotted using myc and FLAG antibodies as inputs for pull-down analysis shown in **c**. **c** The ApoE4-FLAG and ADN-FLAG immunoprecipitates were mixed with purified recombinant CLSP-MycHis (FL-MH) or C-terminally MycHis-tagged CLSP deletion mutants and incubated at 4 °C overnight. The pulled-down precipitates were then immunoblotted using myc and FLAG antibodies. **d** A schematic illustration of the interaction of CLSP, adiponectin, and a CLSP inhibitor.

We then tried to identify the region of CLSP that was responsible for the interaction with ApoE4 and adiponectin, employing several CLSP mutants (Fig. 3a, b). The EHR of CLSP did not bind to ApoE4 while the other four CLSP mutants did (Fig. 3c, ApoE4 beads). On the other hand, ΔN2 did not bind to adiponectin while the other four mutants did (Fig. 3c, adiponectin beads). This result indicates that the core adiponectin-binding site of CLSP is the EHR while the ApoE4-binding site of CLSP is outside the EHR. Collectively, adiponectin enhances and protects the activity of CLSP via the EHR. Another similar pull-down experiment indicated that ApoE4 did not bind to the N-terminal 1-61 amino acid region of CLSP, CLSP(1-61) (Supplementary Fig. 8A, B). Thsese results indicate that adiponectin dominantly acts by binding to the EHR of CLSP that is different from the ApoE-binding region located in the C-terminal region (Fig. 3d). Furthermore, the so-called “collagen-homologous” region of adiponectin on the C-terminal half of adiponectin is the core site of adiponectin for the binding to CLSP (Supplementary Fig. 8C).

### Adiponectin concentrations are reduced in the brains of AD patients and APP/PS1 mice

**Fig. 4:**
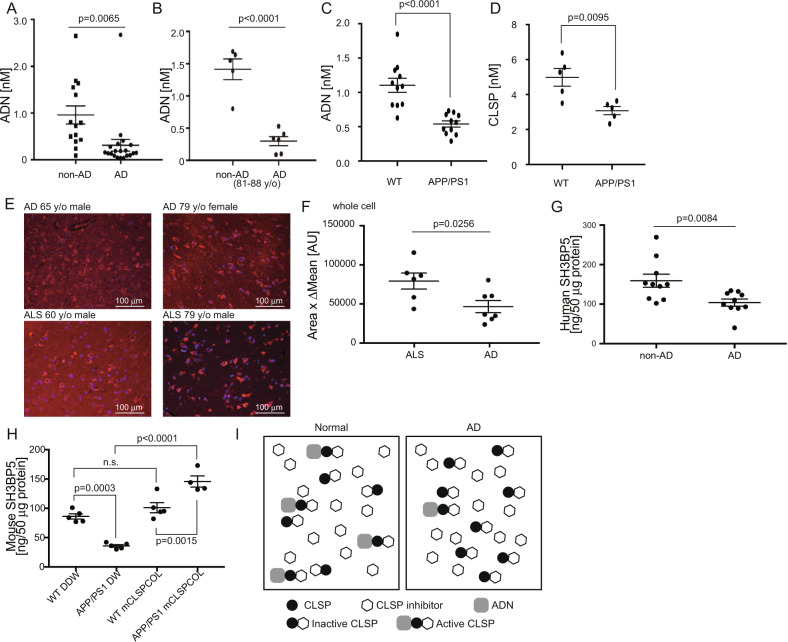
The levels of brain adiponectin and intraneuronal SH3BP5 are reduced in AD patients and aged APP/PS1 mice. **a** CSF adiponectin (ADN) concentrations in AD and non-AD patients (Supplementary Table S1) are shown as dots (*N* = 20 for AD, *N* = 14 for non-AD) with means ± SEM of adiponectin concentrations (AD, 0.31 ± 0.13 nM; non-AD, 0.96 ± 0.19 nM; unpaired *t*-test, *p* = 0.0065). **b** CSF adiponectin concentrations in AD and non-AD patients aged 81–88 years (Supplementary Table S3) are shown as dots (*N* = 6 for AD, *N* = 5 for non-AD) with means ± SEM of adiponectin concentrations (AD, 0.30 ± 0.07 nM; non-AD, 1.41 ± 0.16 nM; unpaired *t*-test, *p* < 0.0001). **c** Adiponectin concentrations in the ISFs of aged male wt littermate and APP/PS1 mice (16-month-old; *N* = 11 for each group) are demonstrated as dots with means ± SEM of adiponectin concentrations (wt, 1.10 ± 0.10 nM; APP/PS1, 0.54 ± 0.05 nM; unpaired *t*-test, *p* < 0.0001). **d** CLSP concentrations in the ISFs of aged male wt littermate and APP/PS1 mice (16-month-old; *N* = 5 for each group) are demonstrated as dots with means ± SEM of mouse CLSP concentrations (wt, 4.99 ± 0.51 nM; APP/PS1, 3.08 ± 0.24 nM; unpaired *t*-test, *p* = 0.0095). **e** Outer pyramidal layers of temporal or occipital lobes from two AD patients (65-year-old, male; 79-year-old female) and two ALS patients (66-year-old, male; 79-year-old, male) were immunostained with the antibody to SH3BP5. Scale bars, 100 μm. The specificity of the SH3BP5 antibody was confirmed as shown in Supplementary Fig. S12A. **f** Immunofluorescence intensities in sections of outer pyramidal layers of temporal or occipital lobes from AD patients and ALS patients are shown as dots (*N* = 7 for AD, *N* = 6 for ALS) with mean ± SEM of immunofluorescence intensities (AD, 46564 ± 7737 arbitrary unit; ALS, 79225 ± 10305 arbitrary unit; unpaired *t*-test, *p* = 0.0256). **g** SH3BP5 concentrations in cell-broken lysates, derived from temporal cortices of AD and non-AD patients listed in Supplementary Tables S6 and S7, were measured using the SH3BP5 ELISA system. Amounts of SH3BP5 in 50 μg of lysates of AD and non-AD patients are shown (*N* = 10 for each group) with their means ± SEM (AD, 103.9 ± 9.0 ng; non-ADs, 159.4 ± 16.5 ng; unpaired *t*-test, *p* = 0.0084). **h** Distilled water (DW) or 5 nmol of mouse CLSPCOL was subcutaneously injected into aged male APP/PS1 and wt littermate mice (16-month-old; *N* = 5 for each group). Twenty-five hr after the injection, mice were sacrificed for the measurement of SH3BP5 concentrations. Amounts of SH3BP5 in 50 μg of cell-broken lysates are shown with their means ± SD (wt+DDW, 88.2 ± 4.5 ng; APP/PS1+DDW, 35.8 ± 2.2 ng; wt+CLSPCOL, 101.0 ± 8.6 ng; APP/PS1+CLSPCOL, 145.8 ± 9.5 ng; one-way ANOVA followed by *post hoc* Tukey test). **i** Schematic diagrams for molecular ratios of CLSP, adiponectin, and a CLSP inhibitor in AD vs normal states.

We next measured the levels of adiponectin in the CSFs, derived from autopsied AD and non-AD cases (Fig. 4a, b, Supplementary Fig. 9, Supplementary Tables 1 and 2) and in the interstitial fluids (ISFs) in the brains of aged male APP/PS1 mice (16-month-old) (Fig. 4c). The levels of CSF adiponectin were lower in AD patients than in non-AD patients (Fig. 4a; Supplementary Table 2). The mean ± SEM concentration of CSF adiponectin in AD cases was 0.31 ± 0.13 nM whereas that in non-AD cases was 0.96 ± 0.19 nM (unpaired *t*-test, *p* = 0.0065). Because the average age of AD patients was smaller than that of non-AD patients (78.5 vs. 86.3 years old) (Supplementary Table 2), CSF adiponectin levels of only patients aged 81–88 years old were compared (Supplementary Table 3), to exclude the effect of the age imbalance between AD and non-AD groups on the result. The adiponectin level was found to be markedly reduced in the CSFs of AD cases aged 81–88 years old (AD, 0.30 ± 0.07 nM; non-AD, 1.41 ± 0.16 nM; unpaired *t*-test,  *p* < 0.0001) (Fig. 4b, Supplementary Table 3). Furthermore, there was no significant correlation between age and CSF adiponectin concentration (correlation coefficient = 0.0055) (Supplementary Fig. S10). Consistently, adiponectin levels were found to be reduced in ISFs in the cortices of APP/PS1 mice (Fig. 4c). Adiponectin concentrations in ISFs of wt and APP/PS1 mice were 1.10 ± 0.10 and 0.54 ± 0.05 nM, respectively (unpaired *t*-test, *p* < 0.0001). Unexpectedly, CLSP concentrations were also found to be reduced in ISFs of the cortices of APP/PS1 mice (Fig. 4d). However, these levels were much higher than the minimal concentration of CLSP (0.5 nM) that is required to suppress the AD-linked neurotoxicity completely in vitro.

CSF ApoE concentrations were also measured to be 259.5 ± 28.3 and 178.3 ± 23.5 nM for the same AD and non-AD cases, respectively (Supplementary Fig. 11A, Supplementary Table 1). Although CSF ApoE concentrations were significantly lower in non-AD cases than in AD patients (unpaired *t*-test, *p* = 0.0309), they were higher than those of CLSP concentrations in the CSF^16^. ISF ApoE concentrations of wt and APP/PS1 mice ranged 21–34 nM (Supplementary Fig. 11B), which were higher than those of ISF CLSP concentrations (Fig. 4d).

### SH3BP5, the intraneuronal effector of the CLSP signaling, is reduced in neurons of AD patients and APP/PS1 mice

We then quantified the intensity of the intraneuronal CLSP-mediated signaling by measuring the levels of intraneuronal SH3BP5, the effector of the humanin/CLSP signaling, the levels of which are transcriptionally regulated by the humanin/CLSP signaling^31^. Immunohistochemical analysis showed that the levels of intraneuronal SH3BP5 were significantly reduced in neurons of AD cortices, compared with those of amyotrophic lateral sclerosis (ALS) cortices (unpaired *t*-test, *p* = 0.0256) (Fig. 4e, f; Supplementary Fig. 12; Supplementary Tables 4 and 5). Although the average age of AD patients was higher than that of ALS patients (78 vs. 69 years old), the aging did not seem to affect the level of SH3BP5, because the level of SH3BP5 was not significantly lower in older persons (equal to or older than 71 years) than in younger persons (younger than 71 years) (unpaired *t*-test, *p* = 0.633) (Supplementary Fig. 13). In addition, ELISA analysis indicated that the SH3BP5 levels were significantly reduced in cell-broken lysates that were derived from the temporal lobes of autopsied AD patients than those from the non-AD patients (unpaired *t*-test, *p* = 0.0084) (Fig. 4g; Supplementary Fig. 14; Supplementary Tables 6 and 7). Similarly, the SH3BP5 levels were reduced in the cerebral cortices of aged APP/PS1 mice, as compared with aged wt mice (one-way ANOVA-Tukey test, *p* = 0.0003) (Fig. 4h, comparing APP/PS1 and wt groups receiving distilled water injection). These results suggest that the downregulation of brain adiponectin results in insufficiency in the intraneuronal CLSP signal in AD (Fig. 4i).

### A fusion protein CLSPCOL is free from the suppression by CLSP inhibitors and passes the blood-brain barrier

The minimal concentration of the N-terminal 61 amino acid region of CLSP, CLSP(1-61), that is required to inhibit AD-related neuronal cell death completely was about 0.5 nM, which is equivalent to that of wt CLSP (Supplementary Fig. 15). As expected from the result above, which indicated that ApoE did not bind to CLSP(1-61) (Supplementary Fig. 8), the CLSP(1-61) activity was not suppressed by ApoE, 14-3-3σ, or calreticulin (Supplementary Fig. 16).

**Fig. 5:**
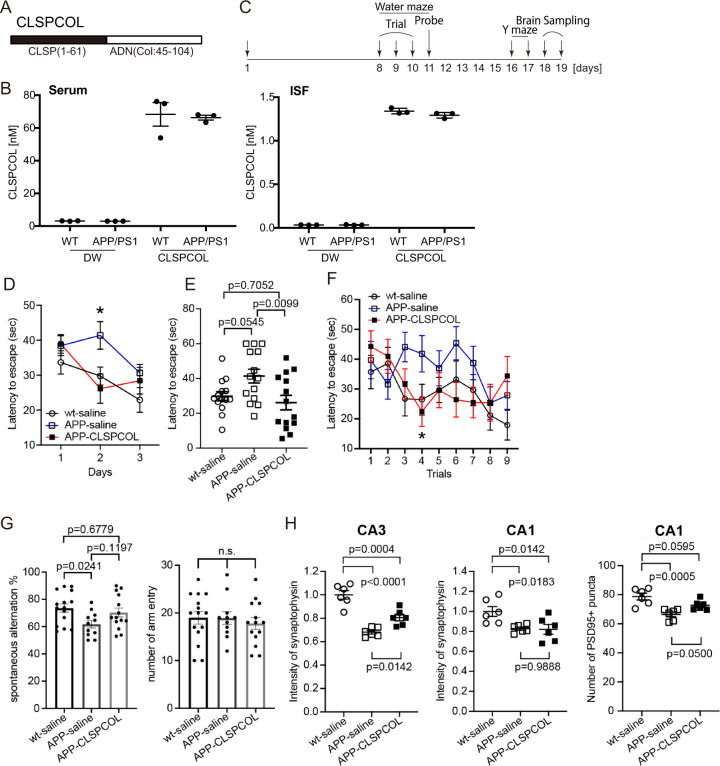
Mouse CLSPCOL alleviates learning impairment and synaptic loss in aged APP/PS1 mice. **a** A schematic diagram of CLSPCOL. **b** Mouse CLSPCOL penetrates the blood-brain barrier efficiently. Twenty-five after a single subcutaneous injection of distilled water or 5 nanomol of mouse CLSPCOL, aged male APP/PS1 and wt littermate mice (16-month-old; *N* = 5 for each group) were sacrificed for the preparation of sera and ISFs. Mouse CLSPCOL concentrations in the sera and ISFs are shown. **c** A schematic illustration of the mouse experimental schedule. **d** Morris water maze trial tests were performed from the 8^th^ to 10^th^ days with three trials per day. Mean ± SEM of latency to escape (time spent to find and escape to the hidden platform) for each group on separate days was shown (days 1-3). Two-way ANOVA followed by *post hoc* Tukey analysis of the three-day results revealed that p was statistically significant (0.0044) for the comparison between APP/PS1-saline and APP/PS1-CLSPCOL groups only for the second day. **e** All raw data on the second day of the water maze test are presented as dots with the mean ± SEM of latency to escape for each group. Statistical analysis was performed using one-way ANOVA followed by *post hoc* Dunnett analysis. **f** Mean ± SEM of latency to escape for each group on each session was shown for three days (total 9 sessions). Two-way ANOVA followed by *post hoc* Tukey analysis of the whole-trial results revealed that *p* was 0.0347 for the comparison between APP/PS1-saline and APP/PS1-CLSPCOL groups, in the fourth trial. **g** From the 16^th^ to 18^th^ day, mice were subjected to the Y maze test. Raw data and mean ± SEM of the alternation percentage for each group (*N* = 15, *N* = 11, and *N* = 14 for wt-saline, APP/PS1-saline, and APP/PS1-CLSPCOL groups, respectively) are shown. Statistical analysis was performed using One-way ANOVA followed by *post hoc* Dunnett analysis. The number of arm entries was counted as a locomotion index on the right panel. n.s. not significant. **h** Coronal hippocampal sections, prepared from three groups (*N* = 6), were immunostained with the monoclonal antibody to synaptophysin, a presynaptic marker (left and central panels), or PSD-95, a postsynaptic marker (right panel). The mean fluorescence intensities of synaptophysin in the 100 × 100 μm areas of the hippocampal CA3 or CA1 regions are presented as a fold change relative to the mean value for wt mice (left and central panels). PSD95-positive puncta were counted in three representative 50 × 50 μm areas in the hippocampal CA1 regions and the average numbers of puncta per area were calculated (right panel). Scatter plot graphs show individual data. Data indicated by bars are the means ± SEM values of six male mice. Statistical analysis was performed using One-way ANOVA followed by *post hoc* Dunnett analysis.

Besides, we found that the collagen-homologous region (45-104 amino acids) was enough to potentiate the CLSP activity (Supplementary Figs. 17 and 18) and possibly protect the CLSP activity from the inhibitors. Considering these findings, we developed a fusion peptide named CLSPCOL that was composed of CLSP(1-61) and the collagen-homologous region (amino acid 45-104) of adiponectin (Fig. 5a). CLSPCOL had more potent CLSP activity than wt CLSP and CLSP(1-61) (the minimal concentration of CLSPCOL that completely suppressed neuronal cell death was 0.1 nM) (Supplementary Fig. 19).

The mouse version of CLSPCOL (mCLSPCOL), consiting of mouse CLSP-1 (1-61) and the collagen-homologous region of mouse adiponectin (amino acids 48-107), must be used for mouse experiments because enough amounts of human CLSP are unable to activate the mouse htHNR, similarly to the human htHNR (5). Chemically synthesized mCLSPCOL suppresses AD-related neuronal cell death in vitro and the minimal concentration of human CLSPCOL and mCLSPCOL that completely suppressed the neuronal cell death was 0.1 nM (Supplementary Figs. 19 and 20). A single subcutaneous injection of 5 nmol of mCLSPCOL resulted in the appearance of mCLSPCOL at the concentrations of 1.34 ± 0.03 and 1.29 ± 0.03 nM in the ISFs of wt littermate and APP/PS1 mice (16-month-old), respectively, at 25 h after injection (Fig. 5b). Although mCLSPCOL concentrations in the ISFs were approximately fifty times smaller than those in sera, they were sufficiently higher than the concentration that was required for mCLSPCOL to suppress the AD-related neuronal cell death completely in vitro (0.1 nM). Another independent experiment suggested that the half-life of mCLSPCOL was more than 24 hr in the CNSs of these mice, which were subcutaneously and intranasally injected with mCLSPCOL (Supplementary Fig. 21).

### CLSPCOL restores the intracellular SH3BP5 levels and alleviates dementia and synaptic loss in the aged APP/PS1 mice

We then examined the effect of injected CLSPCOL. A single subcutaneous injection of 5 nmol of mCLSPCOL resulted in sufficient increase in the SH3BP5 levels in the brains of APP/PS1 mice at 25 hr after injection, as compared with APP/PS1 mice receiving distilled water (DW) (Fig. 4h, comparing APP/PS1 + DW and APP/PS1 + CLSPCOL groups).

We then repeated subcutaneous injection of 5 nmol of mCLSPCOL for the aged male APP/PS1 mice (16 month-old) once per day for 18 or 19 days. These mice were subjected to an independent modified Morris water maze test at the age of 13 months and familiar with this test. A modified Morris water maze test was started on the eighth day and performed for three consecutive days with three trials per day (Fig. 5c). The escape latency in the three groups was not significantly different among three groups on the first day. However, on the second day, the escape latencies of both the wt mice receiving the saline injection and the APP/PS1 mice receiving the mCLSPCOL injection, became shorter than that of APP/PS1 mice receiving the saline injection (Fig. 5d). Statistical analysis (One-way ANOVA-Dunnett) of the second-day’s result indicated that the difference was statistically significant between APP/PS1 mice receiving the mCLSPCOL or the saline injection (*p* = 0.0099) (Fig. 5e). This result suggests that the aged APP/PS1 mice have impaired memory function to learn the location of the platform and mCLSPCOL supplementation restored it. On the third day, the escape latencies in all three groups became similarly shortened possibly because the APP/PS1 mice receiving the saline injections sufficiently learned the place by then and the escape latencies of the other two groups reached the plateau phase on the second day. Comparison of the escape latency in each session (totally 9 trials) indicated that there was a statistically significant difference between APP/PS1-saline and APP/PS1-CLSPCOL groups in the fourth trial (two-way ANOVA-Tukey analysis: *p* = 0.0347) (Fig. 5f). The result of the probe test indicated that three-day education was insufficient for performing the prove test (Supplementary Fig. 22).

To evaluate the short-term working memory, the Y maze test was performed from 16^th^ to 18^th^ day (Fig. 5c). The result indicated that the APP/PS1 mice receiving the saline injections had statistically significant impaired memory function, compared to the wt mice (one-way ANOVA-Dunnett analysis: *p* = 0.0241) and the APP/PS1 mice receiving the mCLSPCOL injections tended to recover the memory function (*p* = 0.1197). The numbers of arm entries were not different between any two groups (Fig. 5g).

Finally, we found that the synaptic loss, monitored by the levels of synaptophysin and PSD95 in the synapses of the hippocampi of the aged APP/PS1 mice^8^, was partially restored by the 18 or 19 day’s injections of mCLSPCOL (Fig. 5h, i). Unexepectedly, total soluble amyloid β levels and soluble amyloid β oligomer levels, but not insoluble (aggregated) amyloid β levels, were reduced by the mCLSPCOL treatment (Supplementary Fig. 23).

## Discussion

An increase in Aβ levels (aggregated fibril forms of Aβ in senile plaques and/or soluble Aβ oligomers) has been regarded as the primary insult in AD for more than 20 years (2). The hyperphosphorylated tau and the abnormalities in the Aβ-unrelated function of amyloid β precursor protein (APP) and presenilins have been regarded as alternative insults. In addition to these insults, we would like to emphasize the existence of AD-suppressing factors, which protect neurons from AD-related insults and the reduction of which is linked to the AD pathogenesis. CLSP likely plays a central role as an AD-suppressing factor (6). In the current study, the therapeutic restoration of the reduced CLSP activity alleviates memory impairment and synaptic loss in aged APP/PS1 mice (Fig. 5). A series of previous studies indicated that potent derivatives of humanin, another agonist of the htHNR, suppressed memory impairment in AD model mice^18,32,33^. Considering these results, we proposed a hypothesis on the AD pathogenesis that two hits were necessary for the onset of AD; an increase in the AD-related neurotoxicity and a decrease in the AD-suppressing activity.

The concentrations of total ApoE were estimated to be much higher (Supplementary Fig. 11A, B)^24–26^ than those of CLSP in the human and murine CNSs (Fig. 4d)^16^. Therefore, in a simple model that is composed of sufficient concentrations of CLSP and much higher amounts of CLSP inhibitors, the CLSP activity is estimated to be null in vivo. However, the current study has revealed that adiponectin enhances the CLSP activity and protects CLSP from CLSP inhibitors in a dominant fashion by binding to the EHR of CLSP even in the presence of higher concentrations of the CLSP inhibitors (Fig. 2). In reality, 1 nM of adiponectin keeps CLSP fully active in the presence of higher concentrations of the CLSP inhibitors (Fig. 2). Thus, adiponectin is regarded as a determinant of the CLSP activity.

Importantly, adiponectin protects CLSP through its collagen-homologous region that may not be essential for the metabolic activity of adiponectin (Supplementary Figs. 17 and 18)^34^. Adiponectin exerts a variety of metabolic functions including glucose and lipid metabolism in the peripheral tissues possibly by binding to canonical adiponectin receptors mainly through its globular domain^35^. It increases insulin signaling, anti-inflammatory, anti-oxidative, and anti-atherogenic functions possibly via two adiponectin receptors on the cell membrane. The transfer of adiponectin across the blood-brain barrier appears to be very limited. The concentration of adiponectin in CSF is nearly 10^3^-fold lower than that in blood serum^36,37^. Given the presence of the canonical adiponectin receptors in the CNS, it is thought that adiponectin functions in the CNS as a regulator of glucose metabolism and a neurogenesis enhancer, and is hypothesized to function as a protective factor. Many studies have provided evidence that the insufficiency of adiponectin or the abnormal regulation of the adiponectin signaling is linked to the onset of AD^38^. The increase or the decrease in serum adiponectin levels^36,37,39^ may be an independent risk factor of AD^40^. Adiponectin levels are downregulated in CSFs of AD patients and correlated with the increase in Aβ levels^37^. Adiponectin knockout mice show an AD-like pathology^41^.

Adiponectin concentrations were reduced in the CSFs of AD patients and the ISFs of aged APP/PS1 mice (Fig. 4a–c), consistent with the results of a previous study^37^. The mean ± SEM concentration of CSF adiponectin in AD patients was 0.31 ± 0.13 nM whereas that in non-AD patients was 0.96 ± 0.19 nM (unpaired *t*-test, *p* = 0.0065) (Fig. 4a–c, Supplementary Table 2). The lowest concentration of the recombinant adiponectin required to maintain full CLSP activity was estimated to be 1 nM in vitro, which may be higher than the mean CSF adiponectin concentration in AD patients. These results suggested that adiponectin that was reduced in the ISFs in the AD brains of AD patients and APP/PS1 mice may be incapable of keeping CLSP sufficiently active in the presence of much higher concentrations of ApoE.

In accordance, the CLSP-mediated protective signaling, correlating with the intraneuronal levels of SH3BP5, was reduced in cortices of AD patients and aged APP/PS1 mice (Fig. 4e–h). Similarly, the level of STAT3 with the phosphorylated 705^th^ tyrosine, an active form of STAT3, which was increased by the humanin/CLSP-mediated protective signaling, was reduced in the hippocampal neurons of AD patients and AD model mice^18^ SH3BP5 is the essential effector of the CLSP-mediated protective activity. Enforced SH3BP5 overexpression suppresses AD-linked neurotoxicity and knockdown of endogenous CLSP expression nullifies the CLSP-mediated suppression of AD-linked neurotoxicity in vitro^31^. SH3BP5 is a direct inhibitor of JNK, which is considered an essential mediator of AD-linked neurotoxicity^42,43^. Activated STAT3 contributes to the upregulation of SH3BP5 transcription^31^. In the current study, therapeutic restoration of the CLSP activity by CLSPCOL alleviates memory impairment and synaptic loss in aged APP/PS1 mice (Fig. 5). This finding strongly supports the idea that insufficiency in the CLSP-mediated neuroprotective signal is directly linked to AD pathogenesis.

Paradoxically, adiponectin has been demonstrated to be upregulated in the sera of AD patients^36,37^ whereas it is reduced in brains (Fig. 4a–c; Supplementary Tables 1–3)^37^. One interpretation is that adiponectin is primarily reduced in the CNS by some AD-related abnormalities and the production of adiponectin in the adipose tissues is secondarily upregulated to recover the reduction. A previous study suggests that adiponectin may be reduced in the CNS of AD patients because adiponectin is immobilized to intraneuronal neurofibrillary tangles containing hyperphosphorylated tau^37^. However, adiponectin was similarly reduced in the brains of the aged APP/PS1 mice (Fig. 4c), in which hyperphosphorylated tau was not formed. Another hypothesis is that the entry of adiponectin to the CNS through the blood-brain barrier is impaired in AD patients and APP/PS1 mice. This hypothesis partially relies on the accumulating evidence suggesting that the function of the blood-brain barrier is compromised in the brains of AD patients and APP/PS1 mice^44^. The exact mechanism remains to be elucidated.

ApoE4 is a major risk factor for AD. The mechanism underlying the ApoE4-mediated increase in the AD risk has been extensively investigated. ApoE4 are hypothesized to exert neurotoxicity by multiple gain-of-function and loss-of-function mechanisms in both Aβ-dependent and independent fashions^45^. In the current study, we have shown that ApoE4 is a slightly stronger inhibitor of CLSP than ApoE3 (Fig. 1c, d). Given the higher concentration of ApoE than that of CLSP in the CNS, it is likely that ApoE3 and ApoE4 may nullify the CLSP activity similarly. However, only free fraction of ApoE that is not lipidated (or not recruited into high-density lipoprotein-like lipid particle) may be used to suppress CLSP. If the concentration of non-lipidated ApoE is comparable to that of CLSP, ApoE may become a determinant of CLSP activity when the levels of adiponectin are reduced. In this case, since ApoE4 suppresses CLSP activity more strongly (Fig. 1c, d), ApoE4 gene carriers become more susceptible to AD insults than non-ApoE4 carriers. Unfortunately, there are no methods to measure the amounts of non-lipidated ApoE to examine the validity of this idea. Additionally, we found that CSF ApoE levels were mildly inceased only in human AD patients (Supplementary Fig. 11A), and ISF CLSP levels were mildly decreased only in APP/PS1 mice (Fig. 4d). Currently, the meaning of these findigs remains unknown. In any case, ApoE levels are apparently much higher than CLSP levels in the CNSs.

To restore the CLSP activity efficciently, we have developed a fusion peptide CLSPCOL, composed of CLSP(1-61) and the collagen-homologous region of adiponectin. CLSPCOL penetrates the blood-brain barrier efficiently (Fig. 5b) and has potent AD-suppressing activity (Supplementary Figs. 19 and 20) that is free from the inhibition by the CLSP inhibitors (Supplementary Fig. 16). CLSPCOL likely retains the activity to potentiate and protect endogenous CLSP through the collagen-homologous region of adiponectin. As an alternative therapeutic method, the administration of ordinary doses of wt CLSP is improper because there are overwhelmingly higher amounts of CLSP inhibitors in the CNS. Peripheral injection of wt adiponectin is also inappropriate because the blood-brain barrier penetration of adiponectin may be impaired in AD.

Unexpectedly, CLSPCOL simultaneously reduced total soluble amyloid β levels and levels of soluble amyloid β oligomers (Supplementary Fig. 23). Given that transgenic overexpression of the *mCLSP-1 (Scarf)* gene attenuated memory impairment and synaptic loss in the aged APP/PS1 mice without affecting the soluble and insoluble amyloid β levels^8^, it is likely that the reduction in amyloid β levels is not the main reason for the CLSPCOL-mediated alleviation of memory impaiment. We speculate that the collagen-homologous region of adiponectin of mCLSPCOL is involved in the CLSPCOL-mediated reduction in soluble amyloid β levels since the knockout of the *adiponectin* gene has been demonstrated to result in the increase in amyloid β levels^41^. Alternatively, considering that chronic administration of a potent humanin analogue reduced amyloid β levels in 3 x FAD mice harboring APP_swe_, tau_P310L_, and PS-1_M146V_, another AD model mouse^32^, we could hypothesize that the signal via the htHNR reduced amyloid β levels simultaneously. The mechanism underlying the CLSPCOL-mediated reduction of amyloid β levels remains to be addressed in the future investigation.

The limitation of this study is that the restoration of the CLSP activity was performed by the introduction of a peptide that contains both EHR and the collagen-homologous region of adiponectin, but not by introduction of a peptide that contains only the collagen-homologous region of adiponectin. Due to technical problems, we have been unable to introduce a peptide that contains only the collagen-homologous region of adiponectin efficiently into the central nervous system. Accordingly, although this study proves that the restoration of the reduced CLSP activity is effective against dementia and synaptic loss of APP/PS1 mice, it does not prove that the reduction in the adiponectin levels is the primary cause of the reduction in the CLSP activity in the barins of APP/PS1 mice. It only offers indirect evidence showing that the adiponectin levels are reduced in the brains of AD patients and APP/PS1 mice to an extent that may result in the reduction in the CLSP activity.

In the current study, adiponectin and SH3BP5 levels were reduced in CNSs of both AD patients and aged APP/PS1 mice (Fig. 4). The CLSPCOL-mediated restoration of the reduced CLSP activity alleviated memory impairment and synaptic loss in APP/PS1 mice (Fig. 5). These data strongly support the idea that the reduced CLSP-mediated protective signaling is essential for the emergence of AD-linked neuronal cell death and dysfunction and its restoration is a reasonable strategy for AD treatment.

## Supplementary information

Supplementary information

Supplementary figures

## Data Availability

All data including raw data are available on request.
