# Supplementary material for: Restoration of the reduced CLSP activity alleviates memory impairment in Alzheimer disease
Source: Transl Psychiatry. 2021 Jan 13;11:44. doi: 10.1038/s41398-020-01168-8 (PMC7806720; doi:10.1038/s41398-020-01168-8)
Supplement: Supplementary file 1 — Supplementary information [file 41398_2020_1168_MOESM1_ESM.docx]

**SUPPLEMENTAR FIGURE LEGENDS**

**Supplementary Figure** **1. 14-3-3 family proteins and secreted calreticulin, but not annexin 2 or 5, suppress the CLSP activity**

(**A-E**) SH-SY5Y cells were transfected with the empty pcDNA3.1/MycHis vector (vector) or pcDNA3.1/MycHis-V642I-APP (V642I-APP). The cells were then cultured in DMEM/F12-10% FBS containing 10 nM of GST-MycHis (GST=MH) or CLSP-MycHis (CLSP-MH) with/without indicated concentrations of BSA, and an 14-3-3 isoform (Results of five isoforms are shown in **A-E** panels). At 24 h after the transfection, the media were replaced with DMEM/F12 with N2 supplement containing the same concentration of GST-MH or CLSP-MH with/without the same concentrations of BSA or the 14-3-3 isoform. At 48 h after the onset of the transfection, cells were harvested to perform trypan blue exclusion cell mortality assays. (**F**) SH-SY5Y cells were transfected with the empty pcDNA3.1/MycHis vector (vector) or pcDNA3.1/MycHis-V642I-APP (V642I-APP). The cells were then cultured in DMEM/F12-10% FBS containing 10 nM of GST-MH or CLSP-MH with/without 100 nM of BSA, calreticulin, annexin 2, or annexin 5. At 24 h, the media were replaced with DMEM/F12 with N2 supplement containing the same concentration of GST-MycHis or CLSP-MycHis with/without 100 nM of BSA, calreticulin, annexin2, or annexin5. At 48 h, cells were harvested to perform trypan blue exclusion cell mortality assays. The cell lysates were immunoblotted using the APP antibody.

**Supplementary Figure 2 Adiponectin does not inhibit the V642I-APP-induced neuronal death or inhibits the CLSP-mediated reduction of the V642I-APP-induced neuronal death**

SH-SY5Y cells were transfected with the empty pcDNA3.1/MycHis vector (vector) or pcDNA3.1/MycHis-V642I-APP (V642I-APP). The cells were then cultured in DMEM/F12-10% FBS plus GST-MH or CLSP-MH with or without indicated stepwise increasing concentrations of recombinant adiponectin (ADN). At 24 h after the transfection, the media were replaced with DMEM/F12 containing N2 supplement plus GST-MH or CLSP-MH with or without indicated stepwise increasing concentrations of recombinant adiponectin. At 48 h after the onset of the transfection, cells were harvested to perform cell viability assays using the WST-8 cell death assay kit or staining with calcein AM, and trypan blue exclusion cell mortality assays. The cell lysates were immunoblotted using the APP antibody.

**Supplementary Figure 3 14-3-3σ levels in human CSF are less than the detection limit**

(**A**)14-3-3σ concentrations in CSFs, derived from eight normal volunteers (CSF#1-8), were measured using the 14-3-3σ ELISA system. The experiment was performed in duplicate. Raw measured numbers for stepwise increasing concentrations of the standard 14-3-3σ (named non-tag sigma concentrations; from 0.195 to 6.25 nM) and CSFs of eight normal volunteers were shown in Abs450 columns. Means of two numbers were then calculated and shown in mean Abs450 columns. PBS was used as the negative control. Del Abs 450nm numbers were obtained by subtracting the PBS number from each mean number. (**B**) A standard correlation line was drawn. The raw data of Del Abs450 nm in (**A**) indicated that the CSF 14-3-3σ concentrations of normal volunteers were under the detection limit (less than zero).

**Supplementary Figure 4. Adiponectin protects CLSP from 14-3-3σ and calreticulin**

(**A, B**) SH-SY5Y cells were transfected with the empty pcDNA3.1/MycHisHis vector (vector) or pcDNA3.1/MycHis-V642I-APP (V642I-APP). The cells were then cultured in DMEM/F12-10% FBS containing 1 nM of GST-MH or CLSP-MH with/without 2 nM of 14-3-3σ (**A**) or 10 nM of calreticulin (**B**) with/without 1 nM of adiponectin (ADN). BSA was used as negative controls. At 24 h, the media were replaced with DMEM/F12 with N2 supplement containing the same combination of proteins. At 48 h, cells were harvested to perform the trypan blue exclusion cell mortality assays. The cell lysates were immunoblotted using the APP antibody.

**Supplementary Figure 5. Adiponectin enhances the CLSP activity**

(**A, B**) SH-SY5Y cells, transfected with the vector or pcDNA3.1/MycHis-V642I-APP, were cultured in media containing indicated concentrations of GST-MH or CLSP-MH with/without indicated concentrations of adiponectin (ADN). At 48 h, the cells were harvested for the trypan blue exclusion and the WST-8 assays. The cell lysates were immunoblotted using the APP antibody.

**Supplementary Figure 6. Trimeric adiponectin has a CLSP-activating effect comparable to wild-type adiponectin**

SH-SY5Y cells were transfected with the vector or pcDNA3.1/MycHis-V642I-APP (V642I-APP). The cells were then cultured in DMEM/F12-10% FBS containing indicated concentrations of GST-MH or CLSP-MH with/without 1 nM of trimeric or wild-type (monomer) adiponectin. Trimeric adiponectin does not multimerize whereas wild-type adiponectin multimerizes to middle-molecular-weight or high-molecular-weight adiponectin. At 24 h, the media were replaced with DMEM/F12 with N2 supplement containing the same combination of proteins. At 48 h, cells were harvested to perform trypan blue exclusion cell mortality assays, the WST-8 cell viability assays, and calcein cell viability assays. The cell lysates were immunoblotted using the APP antibody.

**Supplementary Figure 7. Adiponectin does not compete with ApoE for the binding to CLSP**

(**A**) PBS containing CLSP-MycHis-conjugated sepharose 4B was mixed with/without recombinant apolipoprotein E3 or E4 with/without adiponectin (AND) or annexin2 and incubated at 4°C overnight, followed by extensive washing. The estimated starting concentration of each recombinant protein in the assays was 1 nM. The pulled-down precipitates and inputs including the sepharose 4B beads conjugating CLSP-MH and each recombinant protein, were then subjected to SDS-PAGE, followed by visualization with silver staining. (**B, C**) Scatchard analysis for the measurement of dissociation constants between CLSP and ApoE4 or between CLSP and adiponectin. A standard dose-responsive line was constructed based on the step-wise increasing CLSP-HiBiT concentrations and the corresponding chemiluminescence intensities (AU) in 96-well plates (**B**). For the measurement of CLSP-HiBiT concentrations bound to ApoE4 and adiponectin (ADN), each well in 96-well plates was coated with recombinant ApoE4 or adiponectin at a concentration of 20 pM. Then it was filled with step-wise increasing concentrations of CLSP-HiBiT and incubated at room temperature for two hours, followed by washing and the estimation of CLSP-HiBiT activity by measurement of chemiluminescence using Wallac ARVO^TM^ X5 (Perkin Elmer). This experiment was performed in duplicate and mean data of two wells (Mean) were used for further analysis. From each mean, the mean CLSP-HiBiT activity at the zero concentration was substracted to give real the CLSP-HiBiT concentrations that were bound to adiponectin (ADN) or ApoE4 (Del/MEAN). Then, the concentrations of CLSP-HiBiT that were bound to ApoE4 or ADN (shown as <B>), was estimated, referring to a standard dose-response line (B). Then, unbound concentrations of CLSP-HiBiT (shown as <F>) and B/F were calculated. Dissociation constants were provided by the Scatchard analysis using Prism7 software.

**Supplementary Figure 8. Detailed analysis of the interaction between CLSP and ApoE4 or adiponectin**

(**A, B**) Apolipoprotein E4 binds to the C-terminal region of CLSP. A schematic illustration of deletion mutants of CLSP (**A**). Apolipoprotein E4 (ApoE4) and adiponectin (ADN), C-terminally tagged with FLAG, was overexpressed in F11 neurohybrid cells by transfection. At 24 h after transfection, the F11 cells were harvested for the preparation of cell lysates and the immunoprecipitaion of ApoE4-FLAG and ADN-FLAG using the FLAG antibody (M2 agarose beads). Recombinant CLSP-MycHis (FL-MH) or C-terminally MycHis-tagged CLSP deletion mutants, produced in bacteria, were mixed with ApoE4-FLAG- and ADN-FLAG-containing M2 agarose beads, and incubated at 4°C overnight, followed by extensive washing. The pulled-down precipitates and inputs were then subjected to SDS-PAGE and immunoblot analysis using myc and FLAG antibodies (**B**). (**C**) CLSP binds to the collagen-homologous region of adiponectin. The collagen-homologous region of adiponectin <ADN(Col)>, N-terminally tagged with 6xHis and G (HisG), was produced in bacteria, and CLSP-FLAG was overexpressed in F11 neurohybrid cells by transfection. Purified rcombinant HisG-ADN(Col), and CLSP-FLAG and a control lysates (vector) that were immunoprecipitated with the FLAG antibody, were subjected to SDS-PAGE and immunoblot analysis with the FLAG and HisG antibodies (input; left panel). In parallel, the purified recombinant HisG-AND(Col) were mixed with immunoprecipitated CLSP-FLAG or a control (vector) and incubated at 4°C overnight, followed by extensive washing. The pulled-down precipitates were then subjected to SDS-PAGE and immunoblot analysis with the FLAG and HisG antibodies (Co-IP; right panel).

**Supplementary Figure 9. A standard dose-response line for adiponectin ELISA was constructed by measuring chemiluminescence intensities for stepwise increasing concentrations of recombinant adiponectin**

**Supplementary Figure 10. No correlation between age and CSF adiponectin concentration**

Raw data of adiponectin levels and ages were demonstrated for all subjects in Supplementary table 1 (X axis: ages; Y axis: CSF adiponectin concentrations). Correlation coefficient is 0.0055.

**Supplementary Figure 11. Measurement of the human CSF and mouse ISF ApoE concentrations. ApoE concentrations are much larger than CLSP concentrations.**

**(A)**ApoE concentrations in CSFs, derived from AD patients and controls that are shown in Supplementary tables 1 and 2, were measured using a human ApoE ELISA kit. CSF ApoE concentrations in AD patients and non-ADs was shown as dots (N=20 for AD, N=14 for non-AD) with means ± SEM of adiponectin concentrations (AD, 250.8 ± 28.4 nM; non-AD, 167.3 ± 18.4 nM; unpaired T test, p=0.0309). (**B**) ApoE concentrations in the ISFs of aged male wt littermate and APP/PS1 mice (16-month-old; N=6 for each group) were measured using a mouse ApoE ELISA kit and indicated as dots with means ± SEM of ApoE concentrations (wt, 28.6 ± 1.6 nM; APP/PS1, 29.8 ± 1.0 nM).

**Supplementary Figure 12. Specificity of SH3BP5 antibody and an example of quantification of cellular immunofluorescence intensity**

(**A**) Outer pyramidal layers of temporal lobe from an AD patient (73-year-old, male) were immunostained with mouse IgG (left panel) or the SH3BP5 antibody. (**B**) Mean immunofluorescence intensities of a cell area (x) and a non-cell area surrounding the cell (y) were measured. The relative mean immunofluorescence intensity in the neuron, calculated as (x-y), was multiplied by the neuronal area to estimate the level of SH3BP5 expression in the neuron.

**Supplementary Figure 13. SH3BP5 levels in the neurons were not affected by aging**

SH3BP5 level data of all AD and ALS patients in Figure 4f were divided into two groups based on the age; one group consists of persons with an age of 70 or less and the other with an age 71 or more. Mean ± SD relative intensities of SH3BP5 immunostaining were illustrated for the two groups (57439±14465 arbitrary unit, 65237±7976 arbitrary unit; unpaired *t*-test, p=0.6328, t=0.49, R squared =0.021, degree of freedom= 11, p value by F test=0.24).

**Supplementary Figure 14. A standard dose-response line for SH3BP5 ELISA was constructed by measuring chemiluminescence intensities for stepwise increasing concentrations of reconbinant SH3BP5**

**Supplementary Figure 15. The minimal concentration of CLSP(1-61) that completely suppresses V642I-APP-induced neuronal cell death is 0.5 nM**

SH-SY5Y cells were transfected with the empty pcDNA3.1/MycHis vector (vector) or pcDNA3.1/MycHis-V642I-APP (V642I-APP). The cells were then cultured in DMEM/F12-10% FBS containing indicated concentrations of GST-MH or CLSP(1-61)-MH. At 24 h, the media were replaced with DMEM/F12 with N2 supplement containing the same concentration of GST-MH or CLSP(1-61)-MH. At 48 h, cells were harvested to perform trypan blue exclusion cell mortality, WST8, and calcein assays. The cell lysates were immunoblotted using the APP antibody.

**Supplementary Figure 16. CLSP inhibitors do not inhibit the CLSP(1-61)-mediated suppression of V642I-APP-induced neuronal cell death.**

SH-SY5Y cells were transfected with the empty vector or pcDNA3.1/MycHis-V642I-APP (V642I-APP). The cells were then cultured in DMEM/F12-10% FBS containing 1 nM of GST-MycHis or CLSP(1-61)-MH with 10 nM of BSA(abbreviated as B), ApoE3 (E3), 14-3-3σ (σ), or calreticulin (C). At 24 h, the media were replaced with DMEM/F12 with N2 supplement containing the same concentration of GST-MH or CLSP(1-61)-MH with 10 nM of BSA, ApoE3, 14-3-3σ, or calreticulin. At 48 h, cells were harvested to perform trypan blue exclusion cell mortality, WST8, and calcein assays. The cell lysates were subjected to immunoblot analysis using the APP antibody.

**Supplementary Figure 17. The collagen-homologous domain of adiponectin potentiates the CLSP activity**

SH-SY5Y cells were transfected with the empty vector or pcDNA3.1/MycHis-V642I-APP (V642I-APP). The cells were then cultured in DMEM/F12-10% FBS containing 1 nM or 50 pM of GST-MH or CLSP-MH with 1nM of BSA, adiponectin (FL) or collagen-homologous region of adiponectin (Col). At 24 h, the media were replaced with DMEM/F12 with N2 supplement containing the same combination of proteins. At 48 h, cells were harvested to perform trypan blue exclusion cell mortality, WST8, and calcein assays. The cell lysates were simmunoblotted using the APP antibody.

**Supplementary Figure 18. The minimal concentration of the collagen-homologous domain of adiponectin that enables 50 pM of CLSP to be fully active is 500 pM**

SH-SY5Y cells were transfected with the empty vector or pcDNA3.1/MycHis-V642I-APP (V642I-APP). The cells were then cultured in DMEM/F12-10% FBS containing 50 pM of GST-MH or CLSP-MH with 500 pM of BSA, 250 pM of adiponectin (ADN) or indicated concentration of the collagen-homologous region of adiponectin, ADN(Col). Appropriate concentrations of BSA were further added to make the total concentrations of added proteins to 550 pM. At 24 h, the media were replaced with DMEM/F12 with N2 supplement containing the same combination of proteins. At 48 h, cells were harvested to perform trypan blue exclusion cell mortality, WST8, and calcein assays. The cell lysates were immunoblotted using the APP antibody.

**Supplementary Figure 19. The minimal concentration of human CLSPCOL that is required to suppress neuronal cell death completely is 0.1 nM**

SH-SY5Y cells were transfected with the empty vector or pcDNA3.1/MycHis-V642I-APP (V642I-APP). The cells were then cultured in DMEM/F12-10% FBS containing 1 nM of GST-MycHisG, CLSP(1-61)-MycHisG, or indicated concentrations of bacterially produced recombinant human CLSP(MycHisG)COL (containing the MycHisG tag at the connecting point). At 24 h, the media were replaced with DMEM/F12 with N2 supplement containing the same concentration of reagents. At 48 h, cells were harvested to perform trypan blue exclusion cell mortality assays. The cell lysates were immunoblotted using the APP antibody. Chemically synthesized human CLSPCOL worked similarly.

**Supplementary Figure 20. Chemically synthesized mouse CLSPCOL (mCLSPCOL) has potent cell-death-suppressing activity equivalent to that of human CLSPCOL**

The minimal concentration of mCLSPCOL that is required to suppress mouse neuronal cell death completely is 0.1 nM. F11 cells are the hybrids of rat embryonic day 13 primary cultured neurons and mouse neuroblastoma NTG18 cells. The transient transfection procedure was described previously in detail in reference 5 and an article [

Yamatsuji, T. et al. *Science* **272**, 1349–1352 (1996).]. The cells were transfected with the empty vector or pcDNA3.1/MycHis-V642I-APP (V642I-APP). They were then cultured in media containing indicated concentrations of synthetic mouse CLSPCOL. At 24 h, the media were replaced with Ham's F-12 containing N2 supplement (Invitrogen) containing the same concentration of reagents. At 72 h after the onset of the transfection, cells were harvested to perform trypan blue exclusion cell mortality assays. The cell lysates were immunoblotteds using the APP antibody.

**Supplementary Figure 21. Estimation of half life of mouse CLSPCOL.**

5 nmol of mouse CLSPCOL was subcutaneously injected or 2 nmol of mouse CLSPCOL was intranasally administered [**18**] into aged APP/PS1 and wt littermate mice (16 months old; N=1 for each group). One hr or 25 hr after the administration, mice were sacrificed for the preparation of ISFs. Mouse CLSPCOL concentrations in the ISFs were measured using the single-step mouse CLSPCOL ELISA system.

**Supplementary Figure 22. The probe test in the water maze test on the 11 th day.**

The times spent in one of the four quadrants of the pool are shown. Target, target quadrant; Opposit, opposite quadrant; Right, adjacent right quadrant; Left, adjacent left quadrant.

**Supplementary Figure 23. Soluble amyloid β levels and soluble amyloid β oligomer levels, but not insoluble amyloid β levels, were reduced by mouse CLSPCOL treatment**

(**A-C**) The APP/PS1 and wt littermate mice (16 months old; N=6 for each group) that received once per day subcutaneous injection of saline or 5 nmol of mouse CLSPCOL for 18 days, were sacrificed for the measurement of soluble (**A**), insoluble (aggregated) (**B**), soluble oligomerized (**C**) amyloid β42 concentrations using each ELISA kit. Amounts of Aβ (pmol) per g of brain were presented with means ± SEM (soluble, WT+saline: not detected, APP/PS1+saline: 864.8 ± 155.3, APP/PS1+CLSPCOL: 412.2 ± 90.8, unpaired *t*-test *p*=0.0306; insoluble, WT+saline: not detected, APP/PS1+saline: 6012.0 ± 464.5, APP/PS1+CLSPCOL: 5656.4 ± 470.4; soluble oligomers, WT+saline: 33.9 ±4.7, APP/PS1+saline: 489.2 ± 56.1, APP/PS1+CLSPCOL: 313.8 ± 31.1, unpaired *t*-test *p*=0.021). N.D.: not detected (lower than the detection limit).

**SUPPLEMENTARY TABLES**

**Supplementary table 1**

**The autopsied cases for the examination of CSF adiponectin levels**

| Age | Sex | ApoE | PMD | CERAD stage or Diagnosis | B & B stage |
| --- | --- | --- | --- | --- | --- |
| 81 | M | 34 | 7.2 | Normal CERAD 1B | I |
| 90 | M | 33 | 7.4 | Normal CERAD 1A | II |
| 88 | M | 23 | 17.3 | Normal CERAD 1A | I |
| 86 | M | 34 | 6.1 | Normal CERAD 1A | III |
| 90 | M | 33 | 4.0 | Normal CERAD 1B | II |
| 86 | M | 33 | 16.3 | Normal CERAD 1A | I |
| >90 | M | 34 | 7.7 | Normal CERAD 1B | I |
| >90 | M | 33 | 22.3 | Normal CERAD 1A | III |
| 90 | M | 23 | 3.7 | Normal CERAD 1A | III |
| >90 | F | 23 | 5.0 | Normal CERAD 1B | II |
| 72 | F | 33 | 30.0 | Normal CERAD 1B | II |
| >90 | F | 23 | 5.2 | Normal CERAD 1B | III |
| 85 | F | 33 | 13.0 | Normal CERAD 1A | II |
| 80 | F | 33 | 16 | Normal CERAD 1A | III |
| 73 | M | 33 | 9.4 | Possible AD | III |
| 79 | M | 24 | 6.5 | Possible AD | III |
| 82 | M | 34 | 1.3 | AD | V |
| 83 | M | 33 | 2.0 | AD | V |
| 73 | M | 34 | 22.3 | AD | V |
| 76 | M | 34 | 7.0 | AD | V |
| 80 | M | 44 | 6.5 | AD | V |
| 71 | M | 34 | 6.5 | AD | V |
| 78 | M | 34 | 8.0 | AD | V |
| 85 | M | 34 | 23.5 | AD | V |
| 75 | M | 34 | 12.2 | AD | V |
| 77 | M | 33 | 10.7 | AD | V |
| 83 | M | 33 | 12.8 | AD | V |
| 79 | F | 44 | 16.2 | AD | V |
| 80 | F | 33 | 16 | AD | V |
| 79 | F | 44 | 8.0 | AD | V |
| 77 | F | 44 | 35.4 | AD | V |
| 81 | F | 44 | 14.7 | AD | V |
| 84 | F | 34 | 5.9 | AD | V |
| 75 | F | 34 | 8.8 | AD | V |

ApoE: two apolipoprotein E gene alleles, PMD; postmortem duration before autopsy, B&B stage; Braak & Braak stage.

Two possible AD cases were classified into AD cases.

Mean ± SEM ages of total AD and non-AD cases were 78.5±0.9 and more than 86.3±1.4 years old, respectively (unpaired *t* test, *p*<0.0001 if “more than” before ages is considered to be “equal to”). Mean ± SEM PMDs of total AD and non-AD cases were 11.7±1.8 and 11.5±2.1 hours, respectively (unpaired *t* test, *p*=0.952).

**Supplementary table 2**

**Summary of 34 autopsied cases for the measurement of CSF adiponectin levels**

|  | AD | Non-AD | Unpaired *t* test (two-tailed) | | | | *p* value by F test |
| --- | --- | --- | --- | --- | --- | --- | --- |
|  |  |  | *p* | *t* | R squared | degrees of freedom |  |
| n | 20 | 14 |  |  |  |  |  |
| Gender M/F | 13/7 | 9/5 |  |  |  |  |  |
| Age (±SEM) | 78.5±0.9 | 86.3±1.4 | <0.0001* | 4.92 | 0.43 | 32 | 0.20 |
| ApoE4 allele(s) % | 75.0 | 21.4 |  |  |  |  |  |
| PMD (±SEM) hour | 11.7±1.8 | 11.5±2.1 | 0.952 | 0.061 | 0.00017 | 32 | 0.96 |
| CSF adiponectin conc. (±SEM) nM | 0.31±0.13 | 0.96±0.19 | 0.0065 | 2.92 | 0.21 | 32 | 0.34 |

PMD; postmortem duration before autopsy

* *p* is less than 0.0001 if “more than” before ages is considered to be “equal to”. See ages of cases in Supplementary table1.

**Supplementary table 3**

**Summary of 11 subjects with ages of 81-88 in Supplementary table 1**

|  | AD | Non-AD | Unpaired *t* test (two-tailed) | | | | *p* value by F test |
| --- | --- | --- | --- | --- | --- | --- | --- |
|  |  |  | *p* | *t* | R squared | degrees of freedom |  |
| n | 6 | 5 |  |  |  |  |  |
| Gender M/F | 2/4 | 3/2 |  |  |  |  |  |
| Age (±SEM) | 83.0±0.6 | 85.2±1.2 | 0.106 | 1.8 | 0.264 | 9 | 0.22 |
| ApoE4 allele(s) % | 50 | 40 |  |  |  |  |  |
| PMD (±SEM) hour | 10.0±3.5 | 11.9±2.3 | 0.668 | 0.443 | 0.021 | 9 | 0.34 |
| CSF adiponectin conc. (±SEM) nM | 0.30±0.07 | 1.41±0.16 | <0.0001 | 6.73 | 0.83 | 9 | 0.14 |

PMD; postmortem duration before autopsy

**Supplementary table 4**

**The autopsied cases for the measurement of SH3BP5 immunofluorescence intensities**

|  | Age/Sex | CDR |
| --- | --- | --- |
| ALS | 69/F | NE |
|  | 64/F | NE |
|  | 60/M | NE |
|  | 79/M | NE |
|  | 62/M | NE |
|  | 66/M | NE |
| AD | 65/M | 3 |
|  | 79/F | 3 |
|  | 79/F | 3 |
|  | 83/F | 3 |
|  | 55/F | 3 |
|  | 73/M | 3 |
|  | 97/F | 3 |

Sections of outer pyramidal layers of temporal or occipital lobes were derived from autopsied AD and ALS patients. CDR: Clinical dementia Rating, NE: not examined.

Mean ± SEM ages of total ALS and AD patients were 66.7 ± 2.8 and 75.9 ± 5.1 years old, respectively (unpaired *t* test, *p*= 0.158).

**Supplementary table 5**

**Summary of 13 autopsied cases for the measurement of intraneuronal SH3BP5 in outer pyramidal layers of temporal or occipital lobes**

|  | AD | ALS | Unpaired *t* test (two-tailed) | | | | *p* value by F test |
| --- | --- | --- | --- | --- | --- | --- | --- |
|  |  |  | *p* | *t* | R squared | degrees of freedom |  |
| n | 7 | 6 |  |  |  |  |  |
| Gender M/F | 2/5 | 4/2 |  |  |  |  |  |
| Age (±SEM) | 75.9±5.1 | 66.7±2.8 | 0.158 | 1.51 | 0.17 | 11 | 0.16 |
| SH3BP5 levels (±SD) arbitrary unit | 46564±7737 | 79225±10305 | 0.0256 | 2.56 | 0.38 | 11 | 0.62 |

**Supplementary table 6**

**The autopsied cases for the examination of SH3BP5 levels in temporal lobes**

| Age | Sex | ApoE | PMD | CERAD stage or Diagnosis | B & B stage |
| --- | --- | --- | --- | --- | --- |
| >89 | F | 23 | 5.0 | Normal CERAD 1B | II |
| 78 | F | 33 | 33.0 | Normal CERAD 1A | I |
| 82 | F | 33 | 15.5 | Normal CERAD 1B | I |
| 67 | F | 33 | 8.0 | Normal CERAD 1B | I |
| 65 | F | 33 | 13.6 | Normal CERAD 1A | I |
| 72 | F | 33 | 30.0 | Normal CERAD 1B | II |
| 88 | F | 23 | 20.5 | Normal CERAD 1A | I |
| 84 | F | 33 | 23.0 | Normal CERAD 1B | I |
| >89 | F | 23 | 5.2 | Normal CERAD 1B | III |
| 85 | F | 33 | 13.0 | Normal CERAD 1A | II |
| 79 | F | 44 | 16.2 | AD | V |
| 77 | F | 34 | 6.0 | AD | V |
| 80 | F | 33 | 5.2 | AD | V |
| 79 | F | 44 | 8.0 | AD | V |
| 77 | F | 23 | 35.4 | AD | V |
| 81 | F | 44 | 14.7 | AD | V |
| 84 | F | 34 | 5.9 | AD | V |
| 75 | F | 34 | 5.9 | AD | V |
| 74 | F | 44 | 20.7 | AD | V |
| 88 | F | 34 | 8.7 | AD | V |

ApoE: two apolipoprotein E gene alleles, PMD; postmortem duration before autopsy, B&B stage; Braak & Braak stage

Mean ± SEM ages of total AD and non-AD cases were 79.9 ± 2.9 and more than 79.4 ± 1.3 years old, respectively (unpaired *t* test, *p*=0.876 if “more than” before ages is considered to be “equal to”). Mean ± SEM PMDs of total AD and non-AD cases were 12.7 ± 3.0 and 16.7 ± 3.1 hours, respectively (unpaired *t* test, *p*=0.368).

**Supplementary table 7**

**Summary of 20 autopsied cases for the measurement of SH3BP5 levels in the lysates of temporal lobes**

|  | AD | Non-AD | Unpaired *t* test (two-tailed) | | | | *p* value by F test |
| --- | --- | --- | --- | --- | --- | --- | --- |
|  |  |  | *p* | *t* | R squared | degrees of freedom |  |
| n | 10 | 10 |  |  |  |  |  |
| Gender M/F | 0/10 | 0/10 |  |  |  |  |  |
| Age (±SEM) | 79.9±2.9 | 79.4±1.3 | 0.876 * | 0.16 | 0.0020 | 13.9 | 0.032 |
| ApoE4 allele(s) % | 80 | 0 |  |  |  |  |  |
| PMD (±SEM) hour | 12.7±3.0 | 16.7±3.1 | 0.368 | 0.92 | 0.045 | 18 | 0.95 |
| SH3BP5 amounts in temporal lobe lysates (±SEM)  ng/50μg total protein | 103.9 ± 9.0 | 159.4 ± 16.5 | 0.0084 | 2.96 | 0.33 | 18 | 0.085 |

PMD; postmortem duration before autopsy

* *p* is 0.876 if “more than” before ages is considered to be “equal to”. See the ages of cases in Supplementary table2.

For the analysis of ages, unpaired T test with Welch’s correction was employed because the *p* value by F test was lower than 0.05 (0.032).

**SUPPLEMENTARY MATERIALS and METHODS**

**Genes and vectors**

The human CLSP was inserted into the pcDNA3.1/MycHis (Invitrogen, Carlsbad, CA) to generate CLSP-MycHis （CLSP-MH）, human CLSP that were C-terminally tagged with MycHis in mammalian cells [**5**]. Human apolipoprotein (Apo) E3, E4, adiponectin, annexin 2, and annexin 5 cDNAs were inserted into the pHA vector, a CMV promoter-driven expression vector harboring a C-terminally hemagglutinin A (HA)-tag. Mouse V642I-APP cDNAs inserted in the pcDNA3.1/MycHis vector were described previously (**5**). ApoE3, E4 and adiponectin cDNAs were also inserted into the pFLAG vector to generate C-terminally FLAG-tagged proteins.

*Schistosoma japonicum* glutathione S-transferase (GST)-tagged recombinant proteins were generated in bacteria using the pGEX vectors (GE Healthcare Pharma, Tokyo, Japan), as described previously [**5**]. For the generation of C-terminally HiBiT(amino acid: VSGWRLFKKIS)-tagged CLSP, a sense (5’-CCCGGGGTGAGCGGCTGGCGGCTGTTCAAGAAGATTAGCTGAGAATTC-3’) and an antisense (5’- GAATTCTCAGCTAATCTTCTTGAACAGCCGCCAGCCGCTCACCCCGGG-3’) oligonucleotides encoding the HiBiT amino acids sequence were *in vitro* annealed and inserted into the pGEX-2T-CLSP plasmid at the SmaI-EcoRI site.

To make recombinant N-terminally GST-tagged proteins that were C-terminally tagged with MycHisG, the sequence of the pGEX-2T-MycHis vector was mutated to give rise to the C-terminally addition of a glycine residue using KOD-Plus-Mutagenesis Kit (cat. no.: SMK-101, TOYOBO, Tokyo, Japan) with mutagenesis primers (sense: 5’- GGTTGAGAATTCATCGTGACTGACTGACGATCTGCCTCGCGCG-3’, and antisense primer: 5’- ATGATGATGATGATGATGATCCTCTTCTGAGATGAGTTTTTG-3’). A cDNA of the collagen-homologous region (amino acids 45-104) of human adiponectin was amplified by KOD DNA Polymerase (ca. no.: KOD-101, TOYOBO) with a sense primer: 5’- GGATCCATGAGAGGATCGCATCACCATCACCATCACGGGTCC-3’, and an antisense primer: 5’- GAATTCTCAAGGTTCTCCTTTCCTGCCTTGGATTCCCGGAAAGC-3’, and the amplified cDNA was subcloned into the pGEX-2T vector at the BamHI-EcoRI site. A cDNA of the N-terminally MycHisG-tagged collagen-homologous region of adiponectin was amplified by LA Taq polymerase (ca. no.: RR002A, TaKaRa, Tokyo, Japan) with a sense primer 5’- AAGCTTGAACAAAAACTCATCTCAGAAGAGGATCATCATCATCATCATCATGGTATGGGGCATCCGGGCCATAATGGGGCCCCAGGCC-3’, and an antisense primer 5’- GAATTCTCAAGGTTCTCCTTTCCTGCCTTGGATTCCCGGAAAGCC-3’, and subcloned into the pGEX-2T-CLSP(1-61) plasmids to give rise to human CLSP(MycHisG)COL consisting of CLSP(1-61), the MycHisG tag, and the collagen-homologous region (amino acids 45-104) of human adiponectin.

**Recombinant proteins**

GST-CLSP, C-terminally tagged with MycHis (GST-CLSP-MycHis), was expressed in *E. coli* BL-21 at 37°C for 6 h in 1 mM isopropyl-thio-β-D-galactopyranoside (IPTG, Wako Pure Chemicals, Tokyo, Japan). GST-CLSP-MycHis was bound to glutathione sepharose (GE Healthcare Pharma), and the CLSP-MycHis portion was released from the glutathione sepharose by co-incubation in PBS containing thrombin (1 unit/ml) (cat. no.: T6634-100UN, Sigma-Aldrich, St. Lois, MO) at 25°C overnight, as shown previously [16]. Recombinant CLSP deletion mutants [**5**], C-terminally tagged with MycHis, and CLSP-HiBiT were produced in the same way. Recombinant annexin II, annexin V, SH3BP5, CLSP(1-61)-MycHis, 14-3-3σ, the other 14-3-3 proteins, 6xHisG-tagged collagen-homologous region of human adiponectin, and CLSP(MycHisG)COL were made similarly. Recombinant GST-MycHis were expressed in *E. coli* BL-21 at 37°C for 6 h in 1 mM IPTG, bound to glutathione sepharose, released from the glutathione sepharose by co-incubation in the presence of 50 mM glutathione (Sigma-Aldrich, cat no.: G4251-10G), and was dialyzed in PBS. The N-terminally 6xHisG tagged human SH3BP5 was expressed in *E. coli* M15[pREP4] (Qiagen) at 37°C for 4 h in 1 mM IPTG, bound to the Talon Metal Resin (Clontech, Palo Alto, CA), and purified. Eluted recombinant 6xHis proteins were desalted by Zeba Desalting Column (Pierce) and then one-tenth volume of 10×PBS was added to the desalted protein solution. Recombinant human ApoE3 and ApoE4 were purchased from PeproTech (Rocky Hill, NJ) (cat. no.: 350-02 and 350-04). Human adiponectin and trimeric adiponectin (C39A-adiponectin) that does not oligomerize were purchased from BioVendor (Czeck Republic) (cat. no.: RD172029100 and RD172023100).

**Mouse CLSPCOL**

The amino acid sequence of mouse CLSPCOL (mCLSPCOL: mshgftkeevaefqaafnrfdknkdghisveelgdvmkqlgknlpekdlkaliskldtdgdghpghngtpgrdgrdgtpgekgekgdagllgpkgetgdvgmtgaegprgfpgtpgrkgep) is composed of the N-terminal 61 amino acids of mouse CLSP-1, mCLSP(1-61), and the 60-amino-acid-long collagen-homologous region (the 48-107 amino acid region of mouse adiponectin) that is directly fused. Mouse CLSPCOL was chemically synthesized by BoiSynthesis (Lewisville, TX). Its purity is over 85%. Mass-spectrometry analysis using MALDI/TOF was performed to confirm the molecular weight of the product. It was dissolved in sterile Milli-Q water (Millipore, Bedford, MA) for use. It completely suppressed V642I-APP-induced neuronal death in F11 neurohybrid cells at a concentration of 0.1 nM (Supplementary Fig. 20).

**Antibodies**

Rabbit polyclonal antibodies were raised against synthetic peptides conjugated with keyhole limpet hemocyanin or bacterially produced recombinant proteins, and affinity-purified using synthetic peptides or recombinant proteins. Followings are sets of an immunogen and an antibody name. The N-terminal 16-amino-acid peptide of mouse CLSP-1, mCLSP; GST-CLSP-MycHis, GST-CLSP[**5**]; GST-14-3-3σ, GST-sigma; the C-terminal 16-amino-acid peptide of human 14-3-3σ, sigma-C; GST-SH3BP5, SH3BP5.

Ready-made antibodies against indicated peptides and proteins were purchased from the following companies. FLAG epitope (mouse monoclonal, clone M2, cat. no.: 158592-1MG) (horseradish peroxidase conjugated) from Sigma-Aldrich (St. Louis, MO); APP (mouse monoclonal, clone 22C11, cat. no.: MAB348) from Chemicon (Temecula, CA); Myc epitope (mouse monoclonal, cat. no.: R950-25) and HisG (cat. no.: R940-25) from Invitrogen (Carlsbad, CA); HA (hemmaglutinin A) epitope (mouse monoclonal, clone 3F10, cat. no.: 2013819) (peroxidase conjugated) from Roche Diagnostics (Alameda, CA); SH3BP5 (rabbit polyclonal, named Sab; cat. no.: sc-135617) from Santa Cruz Biotechnology (Santa Cruz, CA); SH3BP5 (mouse monoclonal, clone 1D5, cat. no.: H00009467-M02) from Abnoba, (Taipei, Taiwan); mouse adiponectin (goat polyclonal, cat. no.: AF1119) from R & D systems, Inc. (Minneapolis, MN); PSD95 (mouse monoclonal, cat. no.: MABN68, RRID: AB_10807979) from Millipore (San Diego, CA); synaptophysin (mouse monoclonal, cat. no.: D073-3, RRID: AB_592778) from Medical & Biological Laboratories (Aichi, Japan).

**Preparation of lysates for the measurement of Aβ**

The cell-broken lysates, shown above, were used for ELISA of soluble Aβ. The resulting pellets were resuspended in 70% formic acid containing 1 mM phenylmethylsulfonyl fluoride, aprotinin (10 μg/mL), and 1 mM sodium vanadate and sonicated on ice. The suspensions were centrifuged at 135,000 × *g* for 1 h, and the supernatants (insoluble fractions) were neutralized with formic acid neutralization buffer [1 M Tris, 0.5 M Na_2_HPO_4_, 0.05% NaN_3_].

**Cell death assays**

Experimenters were blinded to identifications of transfected vectors in cell-death assays. Neuronal cell death assays related to AD were first performed by Yamatsuji et al.[ *Science* **272**, 1349–1352 (1996)]. SH-SY5Y cells were grown in DMEM/Ham's F12 mixture (DMEM/F12) containing 10% FBS. SH-SY5Y cells were seeded at 2 × 10^5^/well in six-well plates for 12–16 h, transfected with indicated vectors for 3h in the absence of serum, and then cultured in DMEM/F12-10% FBS with/without CLSP and/or CLSP modifiers. At 24 h after the transfection, the media were replaced with DMEM/F12 containing N2 supplement (Invitrogen) with/without CLSP and/or CLSP modifiers. At 48 h after the onset of the transfection, cells were harvested to perform cell viability assays using the WST-8 cell death assay kit (Dojindo, Kumamoto, Japan) or staining with calcein AM (Dojindo), and trypan blue exclusion cell mortality assays (Trypan Blue Test). Transfection efficiency in SH-SY5Y cells was approximately 80%. All cell-death experiments were performed in triplicate (N=3). F11 neurohybrid cells were previously described in detail [Tamatsuji et al., *Science* **272**, 1349–1352 (1996)].

**Immunoblot analysis**

Cells were washed twice with PBS and suspended in 50 mM HEPES (pH7.4), 150 mM NaCl, 0.1% NP-40, and protease inhibitor cocktail Complete (Roche Diagnostics). After freezing and thawing twice, the cell lysate was centrifuged at 15,000 rpm for 10 min at 4°C. The supernatant and pulled-down precipitates were submitted to analysis with standard or Tris-Tricine SDS polyacrylamide gel electrophoresis (SDS-PAGE) and immunoblot analysis. Ten μg of cell lysates per lane were used for direct immunoblot analysis. The immunoblot analysis using the APP antibody simultaneously visualized both exogenously expressed V642I-APP and the endogenous wild-type APPs with various molecular weights. Due to unknown reason, endogenous wild-type APPs were visualized by immunoblot analysis quite differently among experiments (see figures immunoblotted with the 22C11 APP antibody).

**Pull-down analysis**

Conjugation of a recombinant protein to cyanogen bromide-activated Sepharose 4B was performed according to the manufacturer’s instruction (GE Healthcare Pharma). Briefly, 5 mg of a recombinant protein was incubated with 3 ml of cyanogen bromide-activated Sepharose 4B in a coupling buffer (0.1 M NaHCO_3_ containing 0.5 M NaCl, pH 8.3) at 4°C overnight with constant rotation. Recombinant protein-conjugated sepharose was then incubated in a blocking buffer (0.2 M glycine, pH 8.0) for 2 h at room temperature to eliminate non-specific binding. After blocking, sepharose was washed with the coupling buffer, and 0.1M sodium acetate buffer (pH 4) containing 0.5 M NaCl. Conjugated sepharose 4B was stored in the coupling buffer at 4°C.

Lysates from cells overexpressing various protein in mammalian cells were mixed with GST-MycHis- or CLSP-MycHis-conjugated sepharose 4B at 4°C overnight, followed by extensive washing. The pulled-down precipitates and the cell lysates were then subjected to SDS-PAGE and immunoblot analysis or staining with silver (Wako) to see the interaction between CLSP and proteins. In an experiment, recombinant CLSP, one of its deletion mutants (ΔN1, ΔN2, ΔC1, and EHR), C-terminally tagged with MycHis, was produced in bacteria and purified. They were mixed with F11 cell-derived lysates containing ApoE4 or adiponectin, C-terminally tagged with FLAG at 4°C overnight, followed by extensive washing. The washed pulled-down precipitates and the cell lysates were then subjected to SDS-PAGE and immunoblot analysis.
