# Supplementary material for: Restoration of the reduced CLSP activity alleviates memory impairment in Alzheimer disease
Source: Transl Psychiatry. 2021 Jan 13;11:44. doi: 10.1038/s41398-020-01168-8 (PMC7806720; doi:10.1038/s41398-020-01168-8)
Supplement: Supplementary file 2 — Supplementary figures [file 41398_2020_1168_MOESM2_ESM.pdf]

Figure S1

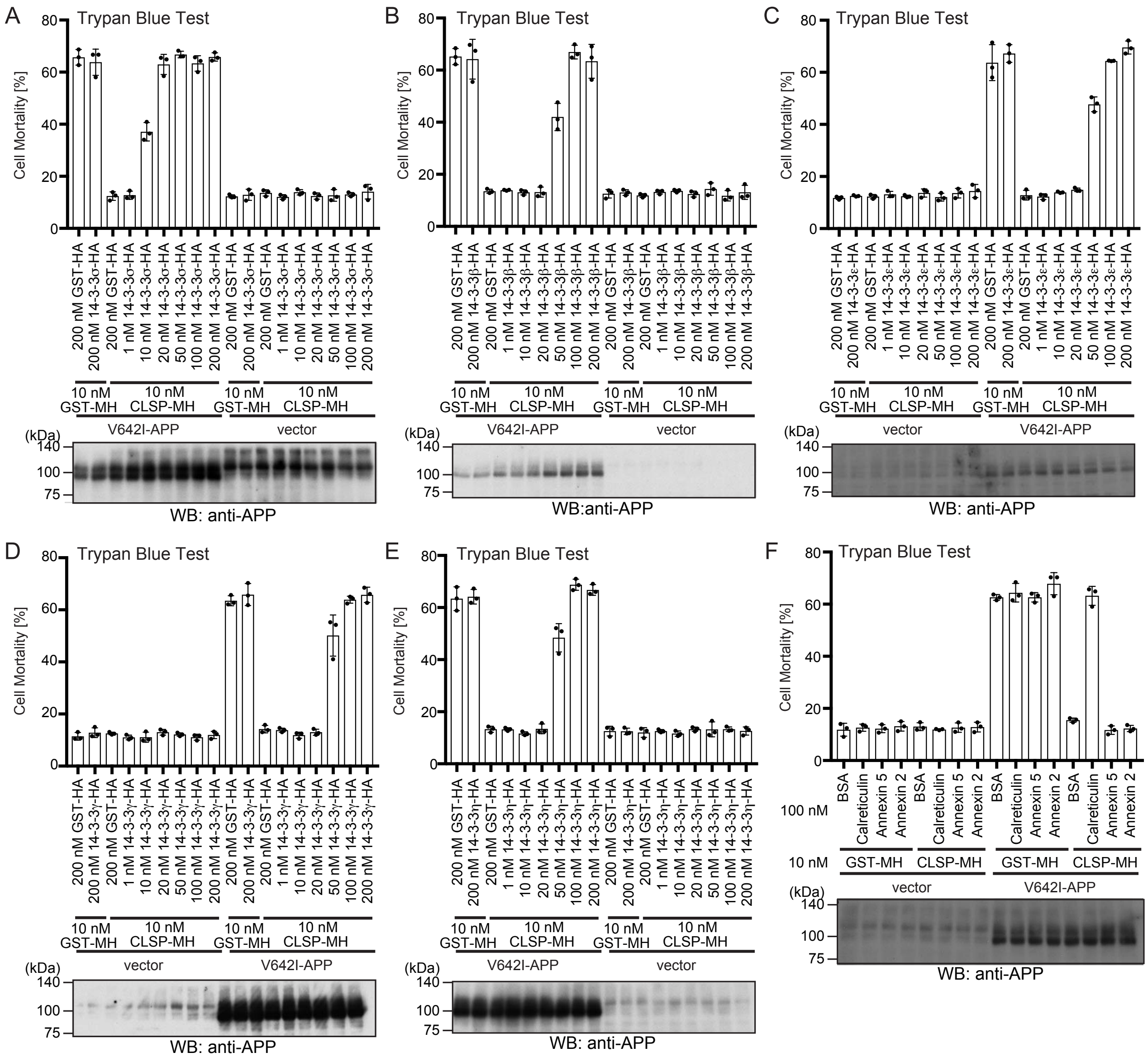

A
Trypan Blue Test
Cell Mortality [%]
200 nM GST-HA
200 nM 14-3-3σ-HA
200 nM GST-HA
1 nM 14-3-3σ-HA
10 nM 14-3-3σ-HA
20 nM 14-3-3σ-HA
50 nM 14-3-3σ-HA
100 nM 14-3-3σ-HA
200 nM 14-3-3σ-HA
10 nM GST-MH
10 nM CLSP-MH
V642I-APP
vector
(kDa)
WB: anti-APP
B
Trypan Blue Test
Cell Mortality [%]
200 nM 14-3-3β-HA
WB:anti-APP
C
Trypan Blue Test
Cell Mortality [%]
200 nM 14-3-3ε-HA
WB: anti-APP
D
Trypan Blue Test
Cell Mortality [%]
200 nM 14-3-3γ-HA
WB: anti-APP
E
Trypan Blue Test
Cell Mortality [%]
200 nM 14-3-3η-HA
WB: anti-APP
F
Trypan Blue Test
Cell Mortality [%]
100 nM
BSA
Calreticulin
Annexin 5
Annexin 2
10 nM
GST-MH
CLSP-MH
vector
V642I-APP
(kDa)
WB: anti-APP

Figure S2

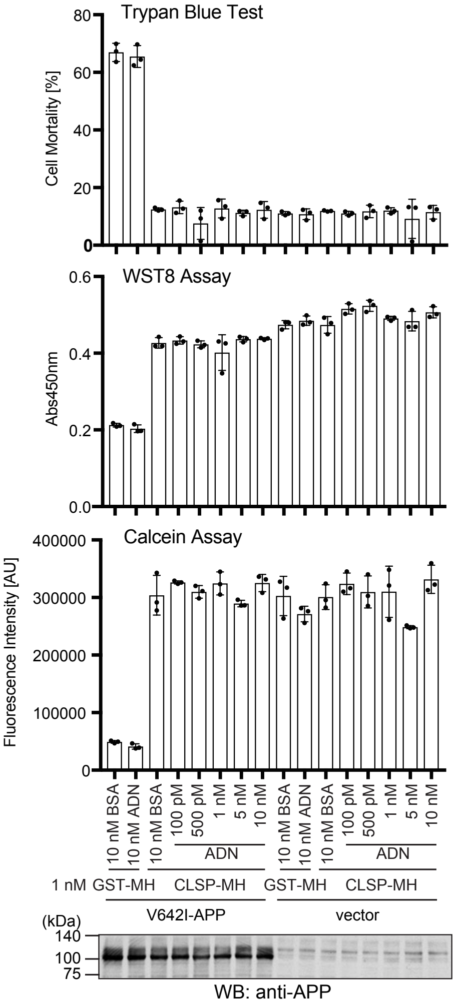

Trypan Blue Test
Cell Mortality [%]
WST8 Assay
Abs450nm
Calcein Assay
Fluorescence Intensity [AU]
10 nM BSA
10 nM ADN
10 nM BSA
100 pM
500 pM
1 nM
5 nM
10 nM
ADN
1 nM GST-MH
CLSP-MH
V642I-APP
vector
(kDa)
140
100
75
WB: anti-APP

Figure S3

A

	[nM]	Abs450nm		Mean Abs450nm	Del Abs450nm	
		1	2			
PBS	0	0.113	0.098	0.106	0.000	
non-tag sigma	0.195	0.123	0.121	0.122	0.017	
non-tag sigma	0.391	0.144	0.149	0.146	0.041	
non-tag sigma	0.781	0.214	0.222	0.218	0.112	
non-tag sigma	1.563	0.377	0.389	0.383	0.278	
non-tag sigma	3.125	0.875	0.899	0.887	0.782	
non-tag sigma	6.250	2.002	2.067	2.034	1.929	
						[nM]
CSF #1		0.086	0.092	0.089	−0.016	X
CSF #2		0.088	0.093	0.090	−0.015	X
CSF #3		0.091	0.097	0.094	−0.012	X
CSF #4		0.084	0.094	0.089	−0.017	X
CSF #5		0.092	0.103	0.097	−0.008	X
CSF #6		0.086	0.092	0.089	−0.017	X
CSF #7		0.091	0.096	0.093	−0.013	X
CSF #8		0.091	0.094	0.092	−0.013	X

B

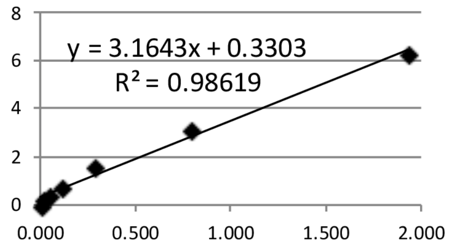

Figure S4

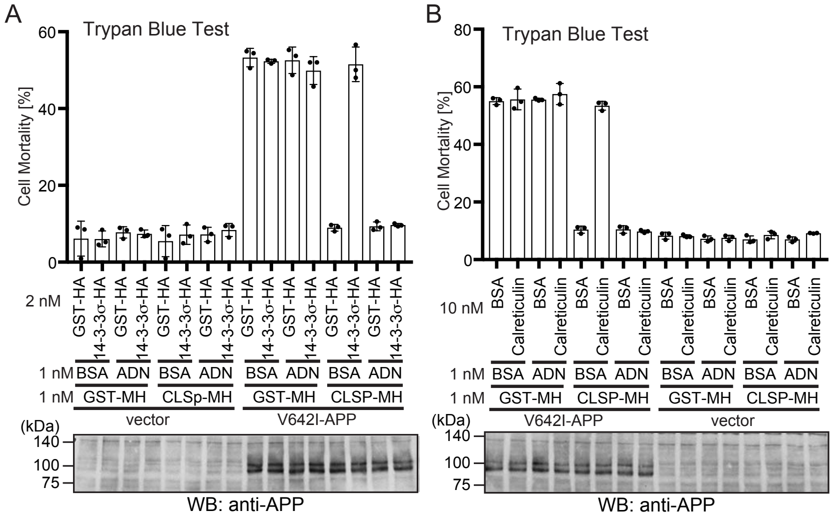

A
Trypan Blue Test
Cell Mortality [%]
2 nM
GST-HA
14-3-3σ-HA
1 nM BSA ADN BSA ADN BSA ADN BSA ADN
1 nM GST-MH CLSp-MH GST-MH CLSP-MH
vector
V642I-APP
(kDa)
140
100
75
WB: anti-APP
B
Trypan Blue Test
Cell Mortality [%]
10 nM
BSA
Calreticulin
1 nM BSA ADN BSA ADN BSA ADN BSA ADN
1 nM GST-MH CLSP-MH GST-MH CLSP-MH
V642I-APP
vector
WB: anti-APP

Figure S5

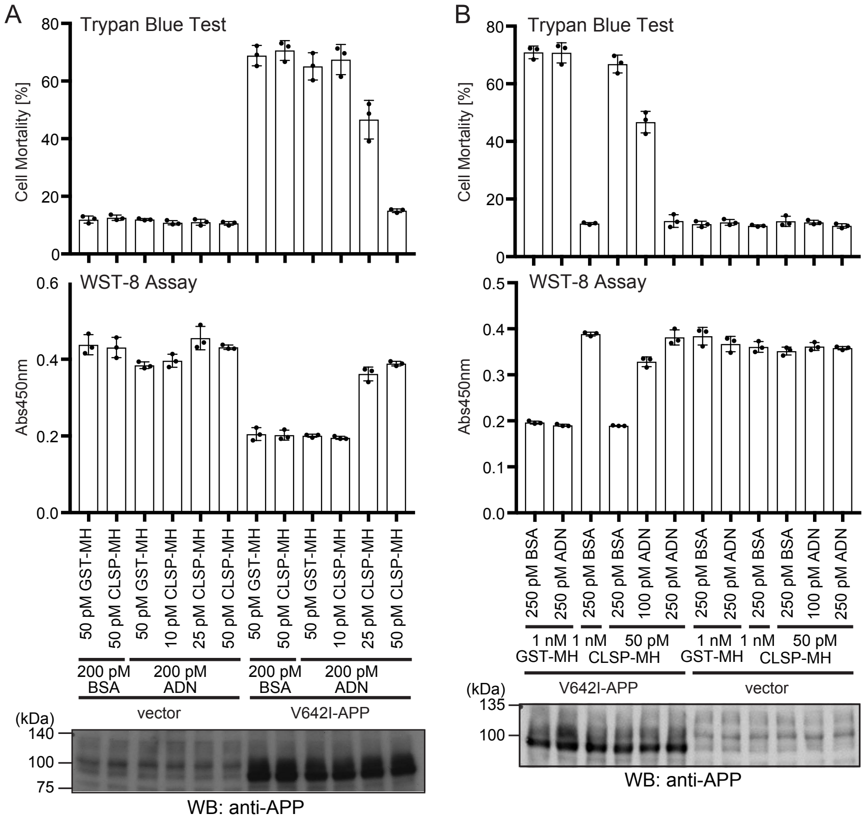

A
Trypan Blue Test
Cell Mortality [%]
WST-8 Assay
Abs450nm
50 pM GST-MH
50 pM CLSP-MH
50 pM GST-MH
10 pM CLSP-MH
25 pM CLSP-MH
50 pM CLSP-MH
200 pM BSA
200 pM ADN
vector
V642I-APP
(kDa)
140
100
75
WB: anti-APP
B
Trypan Blue Test
Cell Mortality [%]
WST-8 Assay
Abs450nm
250 pM BSA
250 pM ADN
100 pM ADN
1 nM GST-MH
1 nM
50 pM
CLSP-MH
V642I-APP
vector
(kDa)
135
100
WB: anti-APP

Figure S6

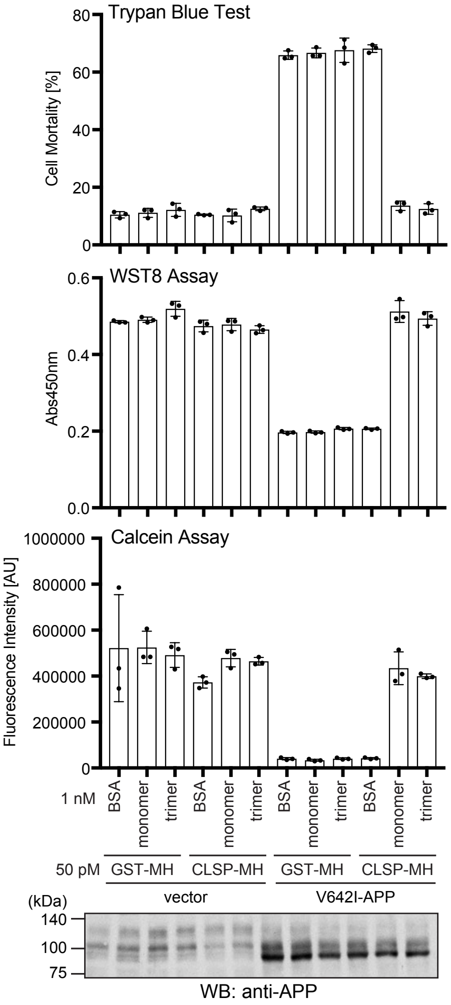

Trypan Blue Test
Cell Mortality [%]
80
60
40
20
0
WST8 Assay
Abs450nm
0.6
0.4
0.2
0.0
Calcein Assay
Fluorescence Intensity [AU]
1000000
800000
600000
400000
200000
0
1 nM
BSA
monomer
trimer
BSA
monomer
trimer
BSA
monomer
trimer
BSA
monomer
trimer
50 pM
GST-MH
CLSP-MH
GST-MH
CLSP-MH
vector
V642I-APP
(kDa)
140
100
75
WB: anti-APP

Figure S7

A

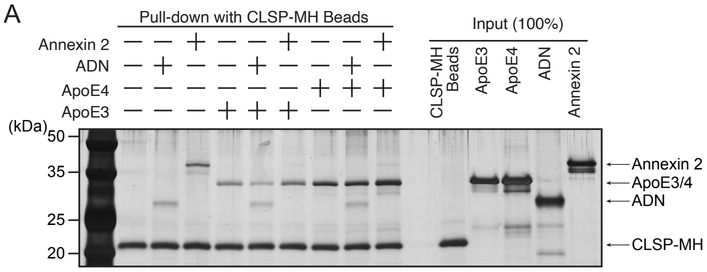

B

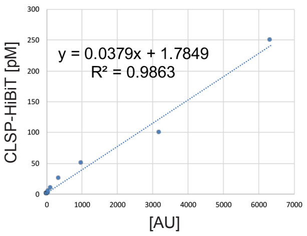

C

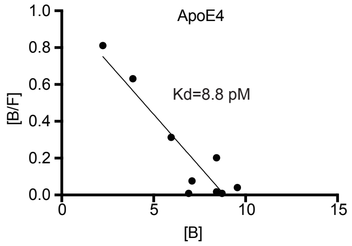

CLSP-HiBiT [pM]	AU		Mean	DelMean	{B}	[F]	B/F
	1	2					
0	10	8	9	0	0		
5	26	16	21	12	2.240	2.760	0.811
10	72	56	64	55	3.869	6.131	0.631
25	122	116	119	110	5.954	19.046	0.313
50	196	172	184	175	8.417	41.583	0.202
100	142	156	149	140	7.091	92.909	0.076
250	196	232	214	205	9.554	240.446	0.040
500	162	206	184	175	8.417	491.583	0.017
750	174	114	144	135	6.901	743.099	0.009
1000	178	206	192	183	8.721	991.279	0.009

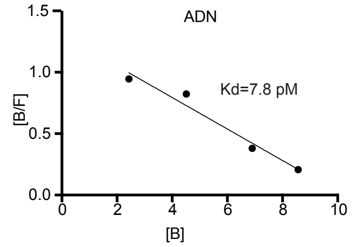

CLSP-HiBiT [pM]	AU		Mean	DelMean	{B}	[F]	B/F
	1	2					
0	8	2	5	0	0		
5	20	24	22	17	2.429	2.571	0.945
10	68	86	77	72	4.514	5.486	0.823
25	124	156	140	135	6.901	18.099	0.381
50	182	186	184	179	8.569	41.431	0.207

Figure S8

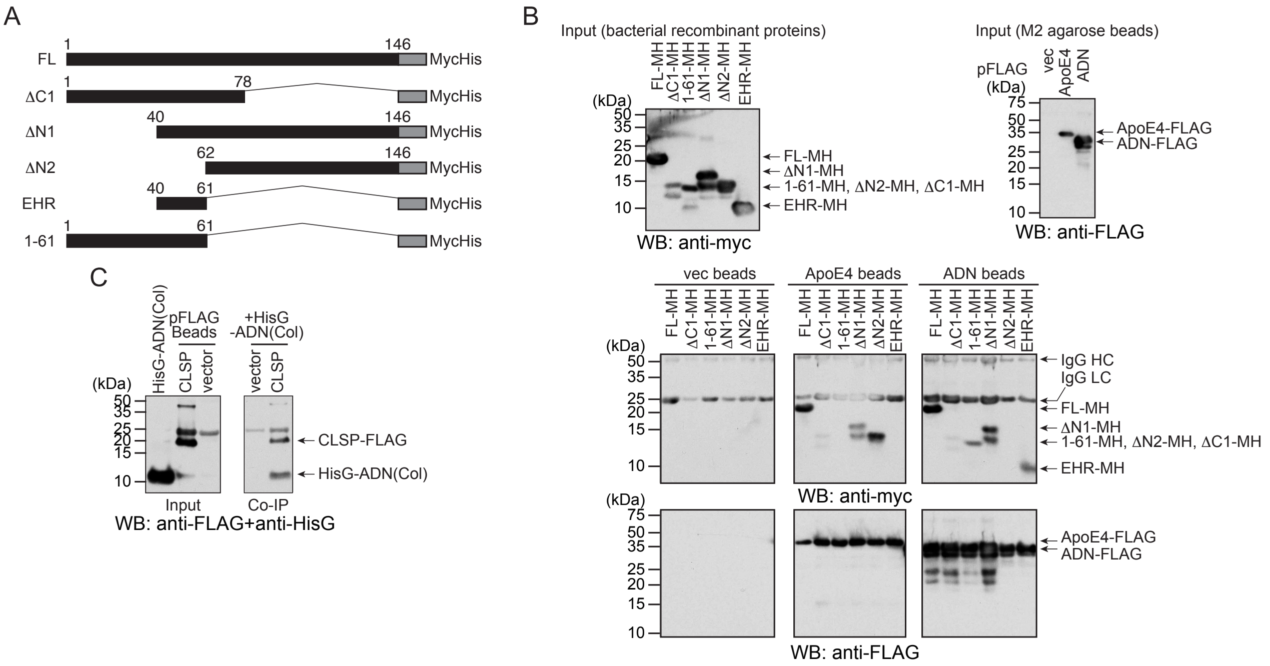

A
FL
1
146
MycHis
ΔC1
78
ΔN1
40
ΔN2
62
EHR
61
1-61
B
Input (bacterial recombinant proteins)
FL-MH
ΔC1-MH
1-61-MH
ΔN1-MH
ΔN2-MH
EHR-MH
(kDa)
1-61-MH, ΔN2-MH, ΔC1-MH
WB: anti-myc
Input (M2 agarose beads)
pFLAG
vec
ApoE4
ADN
ApoE4-FLAG
ADN-FLAG
WB: anti-FLAG
vec beads
ApoE4 beads
ADN beads
IgG HC
IgG LC
C
HisG-ADN(Col)
pFLAG Beads
CLSP
vector
+HisG -ADN(Col)
CLSP-FLAG
Input
Co-IP
WB: anti-FLAG+anti-HisG

Figure S9

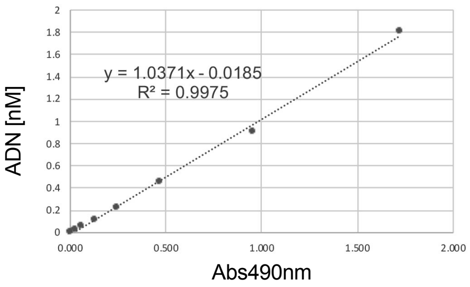
y = 1.0371x - 0.0185
R² = 0.9975
ADN [nM]
Abs490nm
2
1.8
1.6
1.4
1.2
1
0.8
0.6
0.4
0.2
0
0.000
0.500
1.000
1.500
2.000

Figure S10

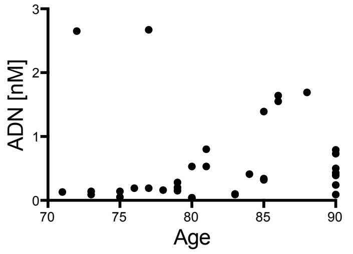

Figure S11

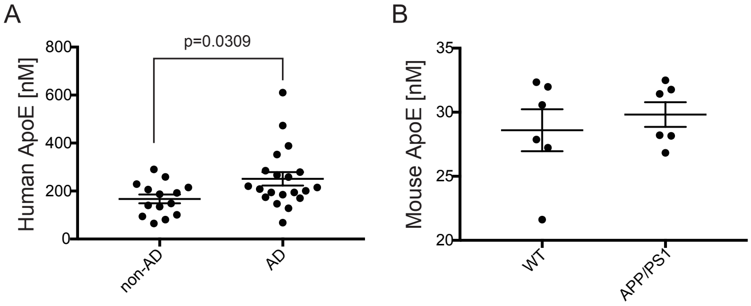
A
p=0.0309
Human ApoE [nM]
800
600
400
200
0
non-AD
AD
B
Mouse ApoE [nM]
35
30
25
20
WT
APP/PS1

Figure S12

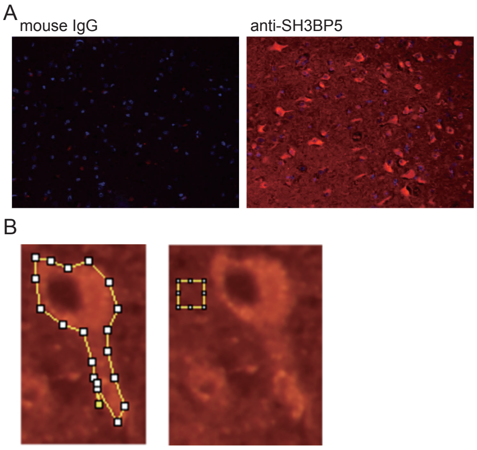

Figure S13

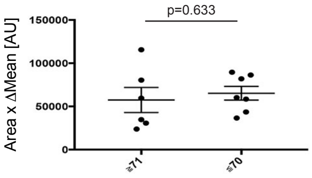

Figure S14

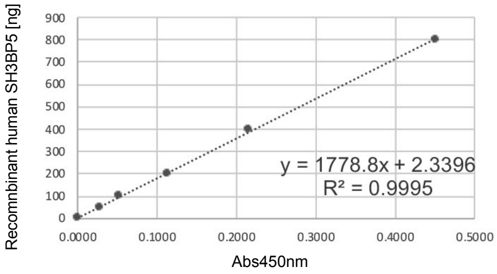

Recombinant human SH3BP5 [ng]
900
800
700
600
500
400
300
200
100
0
0.0000
0.1000
0.2000
0.3000
0.4000
0.5000
Abs450nm
y = 1778.8x + 2.3396
R² = 0.9995

Figure S15

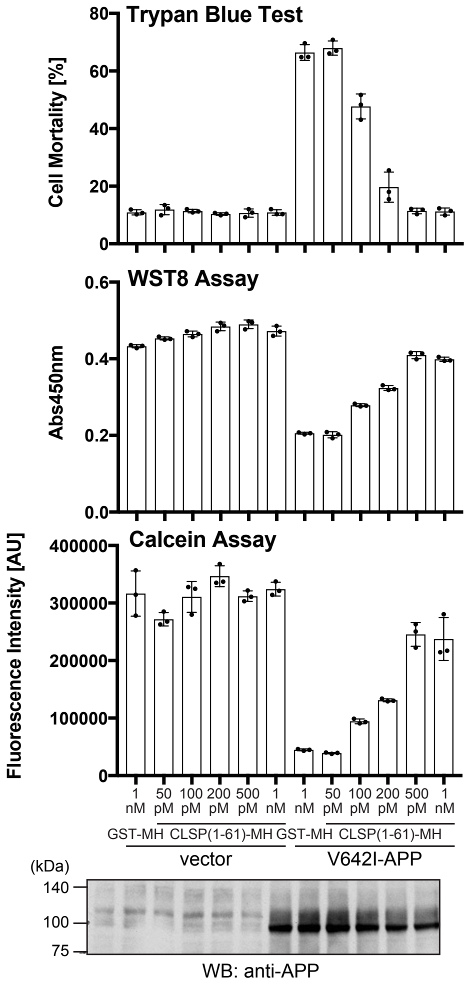

Trypan Blue Test
Cell Mortality [%]
WST8 Assay
Abs450nm
Calcein Assay
Fluorescence Intensity [AU]
1 nM
50 pM
100 pM
200 pM
500 pM
1 nM
GST-MH
CLSP(1-61)-MH
vector
V642I-APP
(kDa)
140
100
75
WB: anti-APP

Figure S16

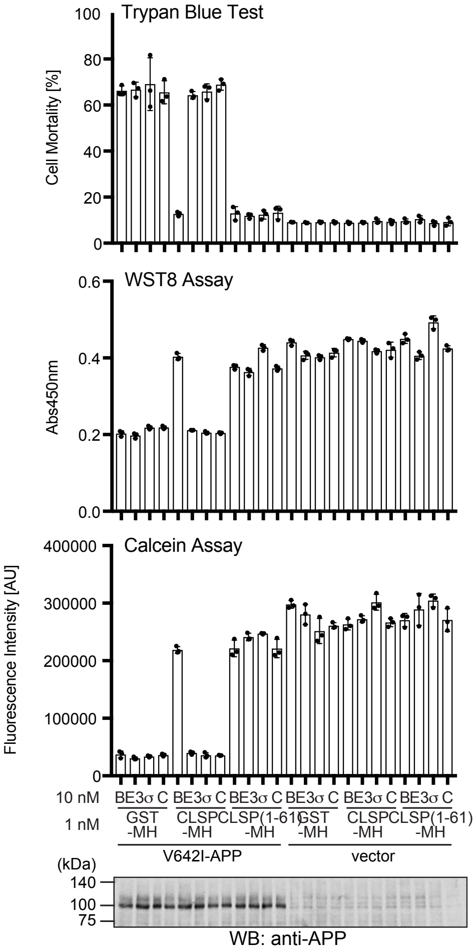
Trypan Blue Test
Cell Mortality [%]
100
80
60
40
20
0
WST8 Assay
Abs450nm
0.6
0.4
0.2
0.0
Calcein Assay
Fluorescence Intensity [AU]
400000
300000
200000
100000
0
10 nM
BE3σ C
1 nM
GST -MH
CLSP -MH
CLSP(1-61) -MH
V642I-APP
vector
(kDa)
140
100
75
WB: anti-APP

Figure S17

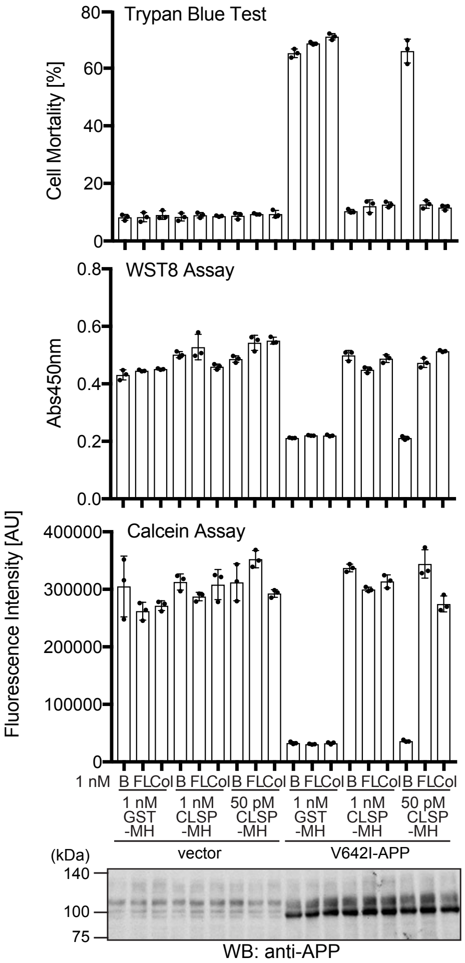

Trypan Blue Test
Cell Mortality [%]
80
60
40
20
0
WST8 Assay
Abs450nm
0.8
0.6
0.4
0.2
0.0
Calcein Assay
Fluorescence Intensity [AU]
400000
300000
200000
100000
0
1 nM B FLCol B FLCol B FLCol B FLCol B FLCol B FLCol
1 nM GST -MH
1 nM CLSP -MH
50 pM CLSP -MH
1 nM GST -MH
1 nM CLSP -MH
50 pM CLSP -MH
vector
V642I-APP
(kDa)
140
100
75
WB: anti-APP

Figure S18

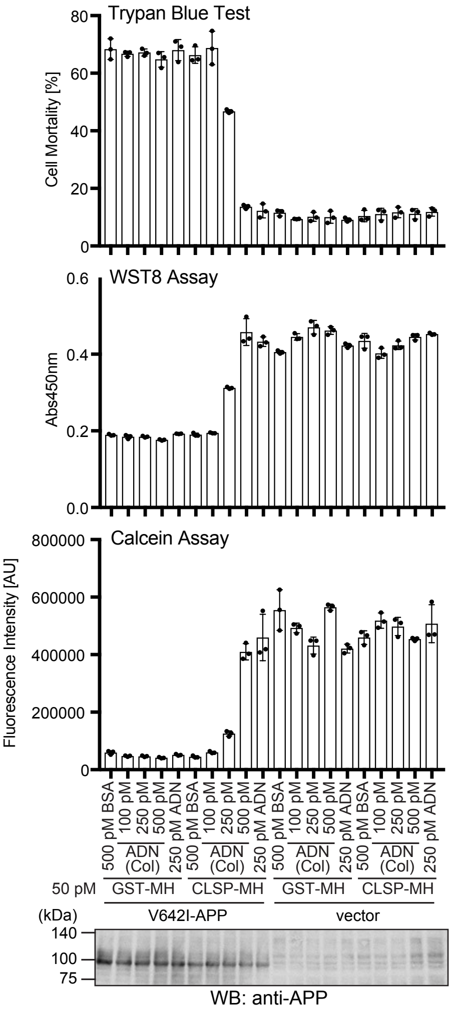

Trypan Blue Test
Cell Mortality [%]
WST8 Assay
Abs450nm
Calcein Assay
Fluorescence Intensity [AU]
500 pM BSA
100 pM
250 pM
500 pM
ADN (Col)
250 pM ADN
50 pM
GST-MH
CLSP-MH
V642I-APP
vector
(kDa)
140
100
75
WB: anti-APP

Figure S19

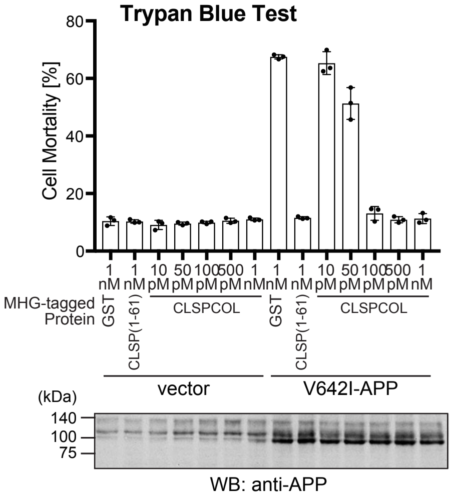
Trypan Blue Test
Cell Mortality [%]
0
20
40
60
80
1 nM
1 nM
10 pM
50 pM
100 pM
500 pM
1 nM
1 nM
1 nM
10 pM
50 pM
100 pM
500 pM
1 nM
MHG-tagged Protein
GST
CLSP(1-61)
CLSPCOL
GST
CLSP(1-61)
CLSPCOL
vector
V642I-APP
(kDa)
140
100
75
WB: anti-APP

Figure S20

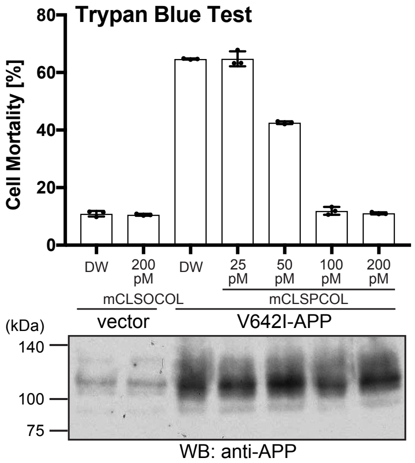

Trypan Blue Test
Cell Mortality [%]
0
20
40
60
80
DW
200 pM
DW
25 pM
50 pM
100 pM
200 pM
mCLSOCOL
mCLSPCOL
vector
V642I-APP
(kDa)
140
100
75
WB: anti-APP

Figure S21

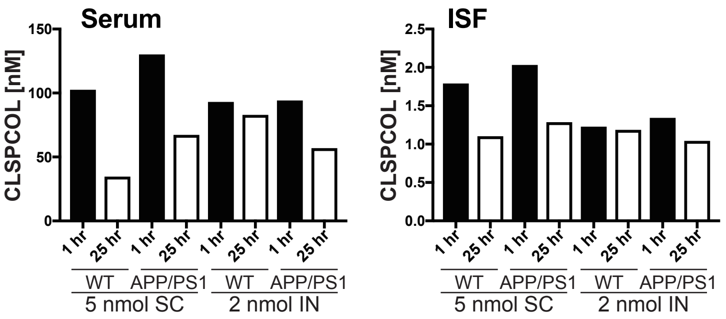
Serum
CLSPCOL [nM]
150
100
50
0
1 hr
25 hr
WT
APP/PS1
5 nmol SC
2 nmol IN
ISF
CLSPCOL [nM]
2.5
2.0
1.5
1.0
0.5
0.0
1 hr
25 hr
WT
APP/PS1
5 nmol SC
2 nmol IN

Figure S22

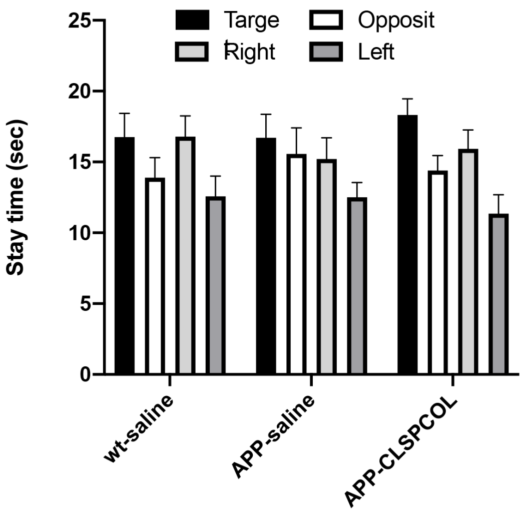

Targe
Opposit
Right
Left
Stay time (sec)
25
20
15
10
5
0
wt-saline
APP-saline
APP-CLSPCOL

Figure S23

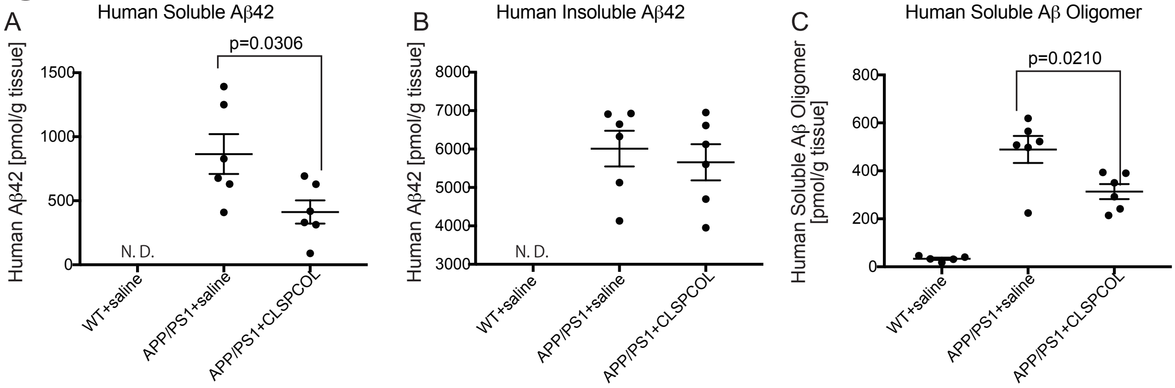
A
Human Soluble Aβ42
Human Aβ42 [pmol/g tissue]
p=0.0306
1500
1000
500
0
N. D.
WT+saline
APP/PS1+saline
APP/PS1+CLSPCOL
B
Human Insoluble Aβ42
Human Aβ42 [pmol/g tissue]
8000
7000
6000
5000
4000
3000
N. D.
WT+saline
APP/PS1+saline
APP/PS1+CLSPCOL
C
Human Soluble Aβ Oligomer
Human Soluble Aβ Oligomer [pmol/g tissue]
p=0.0210
800
600
400
200
0
WT+saline
APP/PS1+saline
APP/PS1+CLSPCOL
